# FXYD5 (Dysadherin) upregulation predicts shorter survival and reveals platinum resistance in high-grade serous ovarian cancer patients

**DOI:** 10.1038/s41416-019-0553-z

**Published:** 2019-08-22

**Authors:** Renata A. Tassi, Angela Gambino, Laura Ardighieri, Eliana Bignotti, Paola Todeschini, Chiara Romani, Laura Zanotti, Mattia Bugatti, Fulvio Borella, Dionyssios Katsaros, Germana Tognon, Enrico Sartori, Franco Odicino, Chiara Romualdi, Antonella Ravaggi

**Affiliations:** 1 ‘Angelo Nocivelli’ Institute of Molecular Medicine, University of Brescia and ASST-Spedali Civili of Brescia, Brescia, Italy; 2 Division of Obstetrics and Gynecology, Department of Clinical and Experimental Sciences, University of Brescia, Brescia, Italy; 3 Department of Pathology, ASST Spedali Civili di Brescia, Brescia, Italy; 4 Division of Obstetrics and Gynecology, ASST Spedali Civili di Brescia, Brescia, Italy; 5 Department of Molecular and Translational Medicine, University of Brescia, Brescia, Italy; 6 Department of Surgical Sciences, Gynecologic Oncology, Città della Salute and S. Anna Hospital, University of Turin, Turin, Italy; 7 Department of Biology, University of Padova, Padova, Italy

**Keywords:** Prognostic markers, Ovarian cancer

## Abstract

**Background:**

High-grade serous ovarian carcinoma (HGSOC) is generally associated with a very dismal prognosis. Nevertheless, patients with similar clinicopathological characteristics can have markedly different clinical outcomes. Our aim was the identification of novel molecular determinants influencing survival.

**Methods:**

Gene expression profiles of extreme HGSOC survivors (training set) were obtained by microarray. Differentially expressed genes (DEGs) and enriched signalling pathways were determined. A prognostic signature was generated and validated on curatedOvarianData database through a meta-analysis approach. The best prognostic biomarker from the signature was confirmed by RT-qPCR and by immunohistochemistry on an independent validation set. Cox regression model was chosen for survival analysis.

**Results:**

Eighty DEGs and the extracellular matrix-receptor (ECM-receptor) interaction pathway were associated to extreme survival. A 10-gene prognostic signature able to correctly classify patients with 98% of accuracy was identified. By an ‘in-silico’ meta-analysis, overexpression of FXYD domain-containing ion transport regulator 5 (FXYD5), also known as dysadherin, was confirmed in HGSOC short-term survivors compared to long-term ones. Its prognostic and predictive power was then successfully validated, both at mRNA and protein level, first on training than on validation sample set.

**Conclusion:**

We demonstrated the possible involvement of FXYD5 and ECM-receptor interaction signal pathway in HCSOC survival and prognosis.

## Background

Epithelial ovarian cancer is the fifth leading cause of cancer death in women and shows the highest mortality rate for gynaecological tumours. In 2018, 22,240 new EOC cases have been predicted in the United States, with 14,070 deaths secondary to this disease.^1^ Epithelial ovarian cancer is a heterogeneous disease accounting for multiple histological variants and clinical behaviours.^2^ Patients harbouring high-grade serous ovarian carcinoma (HGSOC), the most prevalent histotype, are at major risk of cancer related death, since their disease displays an aggressive nature and is usually advanced in stage at diagnosis.^2^ Five-year survival rates for HGSOC patients are around 30–40% world-wide,^3^ however, long-term survivorship is ~15%.^4^ Disparity in prognosis is largely a function of patient age, disease stage and amount of residual tumour after cytoreductive surgery, which are the most important clinical prognostic factors for survival.^5^ Nevertheless, tumours with similar clinicopathological characteristics can have markedly different clinical outcomes. Since prognosis cannot rely exclusively on clinical factors observed at diagnosis, great effort has been made to discover the molecular features of the tumour influencing survival.

The present research aims to identify and validate the best genetically defined survival biomarkers distinguishing long-term HGSOC survivors (overall survival > 7 years) from short-term ones (overall survival < 3 years), by combining high-throughput genomic technology, bioinformatics, classical molecular biology experiments and immunohistochemistry. Our results highlight the potential of FXYD domain-containing ion transport regulator 5 (FXYD5), also called dysadherin or RIC, to predict clinical outcome at the time of first surgery in tissue biopsies of HGSOC patients. FXYD5 is a single-span transmembrane protein belonging to the FXYD family, characterised by a conserved 35-amino-acid signature sequence and involved in the control of ion transport through the interaction with Na^+^/K^+^-ATPase. This protein family also includes FXYD1 (Phospholemman), FXYD2 (ATP1G1), FXYD3 (MAT8), FXYD4 (CHIF), FXYD6 and FXYD7 (RIK).^6^ FXYD5 is able to modulate cellular junctions, to influence chemokine production, and to affect cell adhesion.^7,8^ To the best of our knowledge, this is the first study examining the correlation of FXYD5 protein expression with clinicopathological factors, prognosis and response to chemotherapy in HGSOC patients.

## Methods

### Patients and clinical samples

Sixty-eight cases of surgically resected HGSOCs (divided into training and validation set) were used for the purpose of the study. Inclusion criteria were: original diagnosis of HGSOC based on histologic evidence; stage II to IV; a maximum of 3-years (short-term survivors) or minimum of 7-years (long-term survivors) overall survival (OS) that was defined as the interval between the date of initial surgical resection to death or last follow-up, including monitoring for events of cancer recurrence; availability of representative fresh-frozen tumour specimens (the material containing at least 70% of tumour cells for the RNA extraction); no neoadjuvant chemotherapy.

Patients’ clinicopathological characteristics are described in Supplementary Table S1.

The training set was composed of 39 flash-frozen HGSOC tumour samples (27 short-term and 12 long-term survivors) collected from 2002 to 2008 at the Division of Obstetrics & Gynecologist, ASST Spedali Civili, University of Brescia, Brescia, Italy.

The validation set was composed of 29 flash-frozen HGSOC samples (19 short-term and 10 long-term survivors) enrolled both at ASST Spedali Civili, University of Brescia (from 2005 to 2013) and at the Department of Gynecology Oncology, S. Anna Hospital, University of Torino, Italy (from 2000 to 2004). Patients of both training and validation sets were recruited following a temporal order. With respect to clinical and pathological characteristics (i.e. age, tumour histology and disease stage), training and validation sets were comparable (Supplementary Table S1).

The research was performed following the Declaration of Helsinki set of principles and approved by the Research Review Board—the Ethic Committee—of the ASST Spedali Civili, Brescia, Italy (study reference number: NP1676). Written informed consent was obtained from all enrolled patients.

Progression free survival (PFS) was calculated from the time of surgery until the first clinical recurrence/progression. Cancer recurrence/progression was evaluated by Computed Tomography scan or Magnetic Resonance Imaging. The platinum free interval (PFI) was defined from the last date of carboplatin dose until progressive disease was documented.^9^ HGSOC patients were clinically defined as ‘resistant’, ‘partially sensitive’ and ‘sensitive’ to carboplatin-based chemotherapy on the basis of their PFIs (<6, 6–12 and >12 months, respectively).^9^ Patients known to be still alive at time of analysis or died from another disease were censored at time of their last follow-up.

### Total RNA extraction

Total RNA was extracted and purified from 68 HGSOC biopsies containing at least 70% of tumour epithelial cells. Total RNA extraction and quality control were performed as previously reported.^10^

### Array hybridisation

Thirty-nine HGSOC samples were labelled and hybridised to Affymetrix Human HG-U133 plus 2.0 or Human U133A oligonucleotide microarray chips (Santa Clara, CA, USA) following the manufacturer’s protocols, as previously described.^11^ The gene expression data were deposited in NCBI’s Gene Expression Omnibus^12^ and are accessible through GEO Series accession number GSE131978 (https://www.ncbi.nlm.nih.gov/geo/query/acc.cgi?acc=GSE131978).

### Reverse transcription and real-time quantitative PCR (RT-qPCR)

One microgram of extracted RNA was reverse-transcribed using random hexamers according to the SuperScript TM II protocol (Invitrogen, Thermo Fisher Scientific). The qPCR reactions were performed on CFX96 Touch™ Real-Time PCR Detection System (BIO-RAD Laboratories, Hercules, CA, USA) using the TaqMan Universal PCR master mix and the following TaqMan gene expression assays: Hs00893479_m1 (FXYD5), and HS99999905_m1 (GAPDH). Reaction and thermal cycling conditions were performed as previously reported.^10^ The comparative threshold cycle (Ct) method was used for the calculation of amplification fold, and the delta-delta Ct method was used to obtain relative gene expression values,^13^ normalised using glyceraldehyde-3-phosphate dehydrogenase (GAPDH) reference gene.

### FXYD5 immunohistochemical study of tumour specimens

Tissue microarray (TMA) block was created from 48 formalin-fixed, paraffin-embedded HGSOCs, stored at room temperature in the Department of Pathology at the ASST Spedali Civili/University of Brescia, Italy, was constructed using an automated tissue microarrayer (TMAMaster; 3DHistech, Budapest, Hungary). Representative areas were chosen for sampling from haematoxylin and eosin stained sections of selected HGSOC cases and normal controls. Three 0.6-mm cores have been collected from different areas of each tumour block to overcome tumour heterogeneity and the possible loss of tissue due to cutting. immunohistochemical (IHC) was performed on HGSOC tissue samples from 18 long-term survivors and 30 short-term ones, and on four normal tissues (two ovaries and two fallopian tubes) collected from patients undergoing surgery for benign pathologies.

For IHC, the freshly cut section of the TMA was deparaffinised and rehydrated in graded solutions of ethanol and distilled water. Endogenous peroxidase was blocked by incubation with methanol and hydrogen peroxide 0.03% for 20 min during rehydration. FXYD5 immunostaining was performed using a primary antibody (HPA010817, Polyclonal Rabbit, Sigma-Aldrich, St. Louis, MO, USA) diluted at 1:50 after pre-treatment with microwave in citrate buffer at pH 6.0 (2 cycles of 5 min at 1000 Watt and three cycles of 5 min at 750 Watt). The reaction was revealed using Envision Labelled polymer-HRP anti-Rabbit (Dako, Glostrup, Denmark) followed by diaminobenzidine (DAB, Dako, Glostrup, Denmark). Finally, the slides were counterstained with Meyer’s Haematoxylin. The immunostained TMA section was digitalised by using an Aperio ScanScope CS Slide Scanner Aperio Technologies, (Leica Biosystem, New Castle Ltd, UK) and evaluated for scoring. Two independent observers examined the stained slides in a blinded fashion. Both membranous and cytoplasmic staining was graded for intensity as follows: 0, no reactivity, 1 (weak), 2 (moderate) and 3 (strong). The percentage of positive cells was scored as 0 (0%), 1 (1%-10%), 2 (11%-49%), 3 (50%-89%), or 4 (90–100%). A final score of 0–12 was obtained by multiplying the intensity and percentage scores. Two scales, one for membranous and one for cytoplasmic signal, respectively, were added obtaining a single scale ranging from 0–24, and three total scores were calculated grouping scores 1–6 in total score 1+, scores 7–12 in total score 2+, and scores 13–24 in total score 3+. Digital images were resized by using Adobe Photoshop (Adobe Systems, Inc., San Jose, CA).

### Statistical analysis

#### Microarray data processing and analysis

Raw Affymetrix data (CEL files) were quantified using robust multiarray model^14^ with quantile normalisation. Probes were annotated using custom definition file (CDF) as defined in a previous study.^15^ Combat model^16^ was used to remove batch/platform effect. Differentially expressed genes between long-term and short-term HGSOCs were identify using the Empirical Bayes moderated test.^17^ False Discovery Rate (FDR) was set to 0.1. Gene set enrichment analysis on Gene Ontology terms has been performed using DAVID web tool,^18^ while GraphiteWeb web tool^19^ has been used to run enrichment and topological analyses on Kyoto Encyclopaedia of Genes and Genomes (KEGG) pathways.

#### Discriminant genes on the training set

A penalised logistic regression model^20^ (as implemented in the *penalized* R package) has been used to select the best discriminating genes (between long- and short-term survivors) among those identified as differentially expressed. A first step of leave-one-out cross validation has been used to select the best penalisation parameter on the entire set of variables, and a second step of leave-one-out cross validation was used on the final model to predict its predictive power.

#### In silico validation using curatedOvarianData database

The curatedOvarianData database^21^ available at the Bioconductor platform provides the normalised data of clinically well annotated expression datasets including the ovarian TCGA data.^22^ From the entire curatedOvarianData datasets, we selected HGSOC samples with complete follow-up for PFS and OS, obtaining a panel of seven datasets (GSE17260 *n* = 84, GSE26193 *n* = 79, GSE30161 *n* = 45, GSE49997 *n* = 171, GSE9891 *n* = 239, TCGA array *n* = 481, TCGA RNA-seq *n* = 242) for a total of 1341 samples. Using these publicly available data, we validated the discriminant genes identified in the previous step. To reach this goal, we applied a two-step strategy: (i) we selected long- and short-term survival patients testing expression differences of the gene signature between these two classes; (ii) to overcome possible bias due to the unbalance of the sample sizes of step (i), we performed a survival analysis on the entire cohort of patients using a meta-analysis approach. We decided to select as reliable only genes resulting significant in both conditions. Meta-analysis combination of the *p*-values was obtained using the Fisher method implemented in the metap R package.

#### Statistical analysis on the validation set

The association between FXYD5 log transformed relative gene expression measured by RT-qPCR and clinical variables (including long-term and short-term classes) was assessed using t-test, while the association between FXYD5 protein expression evaluated by IHC staining (coded as score ≤1+, 2+, and 3+) and clinical variables was investigated using Wilcoxon-Mann–Whitney test (in case of numerical clinical variables) or chi-squared (in case of categorical clinical variable). Spearman’s rank correlation was used to estimate the degree of association between microarray and RT-qPCR, and between FXYD5 expression measured by RT-qPCR and IHC staining. For survival analysis, two endpoints (cancer relapse/progression and death for cancer) were used to calculate progression-free survival (PFS) and disease-specific overall survival (OS), respectively. Cox proportional hazard model^23^ was used for survival analysis, while Kaplan–Meier method^24^ was used to draw survival curves. In survival model the average log gene expression (by RT-qPCR) was used as threshold to categorise patients (Low: ≤ 3.30; High: > 3.30). In all analyses, a *p* value < 0.05 was considered as significant. All statistical analyses were performed using the R language.

## Results

### Identification of prognostic gene signature in patients with HGSOC

Eighty mRNAs were found differentially expressed (FDR < 0.1) between the two extreme survivor groups in the training set (27 short-term and 12 long-term survivors): 28 genes (35%) were under-expressed and 52 (65%) were over-expressed in long-OS patients compared to short-OS ones (Supplementary Table S2).

We performed a gene set enrichment analysis on the 80 differentially expressed genes (DEGs) but we did not find any significant Gene Ontology term with FDR < 0.1. In an attempt to identify potential signalling pathways involved in HGSOC survival, we used KEGG database to perform pathway analyses with both enrichment-based (hypergeometric test) and topological-based^25^ approaches. While the first approach tests if the amount of DEGs in a pathway is higher than the proportion that would have been obtained by chance, the second checks also the position of DEGs within pathway-map, giving high priority to the upstream genes of the pathway. Both approaches identified ECM-receptor interaction pathway as highly significant (hypergeometric test FDR = 5.37E-07, SPIA FDR = 2.3E-07).

Then, we looked for drug-target interactions using the Drug Gene Interaction Database.^26^ We detected 10 genes presenting at least one known drug-target interaction among the 34 exact matches provided by the Drug Gene Interaction Database. We report the complete gene list in Supplementary Table S3.

To identify, among the 80 DEGs, those with the highest discriminating potential, we used a penalised logistic regression. A panel of ten prognostic genes (Table 1) was selected by the model as the best discriminating genes. Using cross validation on the training set, this signature was able to correctly predict 98% of patients within their survival class, a significantly higher percentage than that achieved using only clinical variables (ascites, grade, stage, residual tumour and relapse), equal to 84%.

**Table 1:** List of the ten most discriminating genes between long- and short-term survivors

Gene symbol	Gene Name	logFC	*P*-value	Adj *P*
C6orf62	chromosome 6 open reading frame 62	0.653	6.6E-06	0.044
BTN3A3	butyrophilin subfamily 3 member A3	0.855	1.4E-05	0.049
CXCL11	C-X-C motif chemokine ligand 11	1.974	2.9E-05	0.049
DEPTOR	DEP domain-containing MTOR interacting protein	1.486	3.1E-05	0.049
FSTL3	follistatin like 3	−0.528	6.9E-05	0.049
UBE2K	ubiquitin conjugating enzyme E2 K	0.555	9.1E-05	0.049
ANO1	anoctamin 1	0.991	9.7E-05	0.049
FXYD5	FXYD domain-containing ion transport regulator 5	−1.397	9.8E-05	0.049
C8orf33	chromosome 8 open reading frame 33	0.876	1.0E-04	0.049
FDPS	farnesyl diphosphate synthase	0.541	1.3E-04	0.056

*logFC* log fold change of the gene between long-OS and short-OS, *P-Value* uncorrected *p*-value, *Adj. P* FDR-corrected *p*-value

Among these prognostic genes, eight mRNAs were highly expressed in long-term survivors, while the remaining two (FSTL3 and FXYD5) were highly upregulated in short-term survivors.

### Validation of the ten-gene signature in the curatedOvarianData database

The prognostic power of the ten-gene signature was further tested on the curatedOvarianData database. As expected, extreme survivors’ classes were highly unbalanced (GSE17260 63 short *vs* 0 long-OS patients, GSE26193 53 short *vs* 19 long-OS patients, GSE30161 31 short *vs* nine long-OS patients, GSE49997 150 short *vs* 0 long-OS patients, GSE9891 189 short *vs* nine long-OS patients, TCGA array 342 short *vs* 28 long-OS patients, TCGA RNA-seq 157 short *vs* 14 long-OS patients), therefore we decided to define as validated only those mRNAs showing (i) significant differential expression between long-term and short-term survival classes and (ii) significant association to OS using Cox survival models on the entire cohort of patients. Since GSE17260 and GSE49997 do not include long-survival samples, they were not used for step (i).

Using the first criterion, we confirmed the prognostic value of FXYD5 mRNA (meta-analysis *p*-value = 0.007), that resulted overexpressed in short-term compared to long-term survivors. By the second approach, FXYD5 was found associated to OS on the entire curatedOvarianData database (*p* = 0.0002 HR = 1.17 CI95% 1.1–1.3), suggesting its valuable role in prognosis prediction.

### Validation of FXYD5 mRNA by RT-qPCR in the training and validation set

FXYD5 expression was assessed using an independent experimental technique, RT-qPCR, either on the training set and on a third independent cohort.

In the training set, the FXYD5 overexpression in short-term compared to long-term survivors was confirmed as statistically significant (FC = 1.96, *p* = 0.027, *t*-test) (Supplementary Fig. S1A) and strongly correlated with the array values (rs = 0.70; *p* < 0.001).

Then, FXYD5 mRNA expression was further validated in a third and independent cohort of 29 tissue samples (19 short-term and 10 long-term survivors) (Supplementary Table S1). A significant FXYD5 overexpression was further confirmed in short-term compared to long-term survivors (FC = 2.68, *p* = 0.001, *t*-test) (Supplementary Fig. S1B).

### Immunohistochemical validation of FXYD5 protein expression in HGSOC patients

**Fig. 1:**
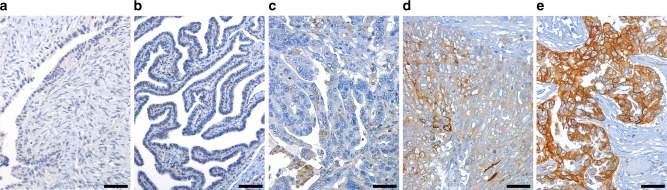
Representative FXYD5 expression by IHC. Negative signals are detectable in normal ovary (**a**) and normal tube (**b**), whereas positive membrane and cytoplasmic immunostaining scored 1+ (**c**), 2+ (**d**) and 3+ (**e**) are shown for HGSOC. Original magnification: ×100 (**a**, **b** scale bar 200 μm); ×200 (**c–e** scale bar 100 μm)

FXYD5 protein expression was analysed by IHC in a cohort of 48 HGSOC tissue specimens (18 long-term and 30 short-term survivors) matched to flash-frozen tissue biopsies examined by RT-qPCR (38 samples belonging to the training set and 10 samples belonging to the validation set). Moreover, four normal tissue samples (two fallopian tubes and two ovaries) were evaluated. Both normal ovaries and fallopian tubes showed a negative FXYD5 immunostaining (Fig. 1a, b), or cytoplasmic/or cytoplasmic staining pattern.

Among long-term survivals, 14 out of 18 (78%) showed weak immunostaining for FXYD5 (Fig. 1c), while the remaining 4 samples (22%) had a moderate signal (Fig. 1d). On the contrary, in the short-term survival group, only 11 out of 30 cases (36%) were detected weakly positive, 11 samples (36%) were scored as moderate, and the remaining eight (28%) were strongly positive (Fig. 1e).

Overall, our results showed a significant correlation between FXYD5 mRNA and protein expression data (rs = 0.48, *p* < 0.001).

### FXYD5 genomic alterations

We analysed the genomic alterations of FXYD5 through the mutational data available in the cBioPortal for Cancer Genomics tool (www.cbioportal.org). FXYD5 showed DNA copy number gain at chromosome 19q13.12 in 26 out of 316 (8.2%) HGSOC samples, but absence of somatic point mutations in all HGSOC samples analysed from the TCGA study. Alteration in the FXYD5 gene expression level occurred in 7.6% of these patients and a linear positive relationship between copy number variation (CNV) and mRNA expression was found (*r* = 0.24, *p* < 0.001) (Supplementary Fig. S2).

When we analysed FXYD5 CNV in cancer cell lines available in the Cancer Cell Lines Encyclopedia (CCLE) Database of the Broad Institute,^27^ we found that FXYD5 showed a general higher CNV score in ovarian cancer cell lines than all others tumour types. In particular, COV318, COV504 and OVCAR4 showed the highest CNV increase (Supplementary Fig. S3).

### Correlations between the expression of FXYD5 and clinicopathological factors

In the entire patients’ cohort evaluated in this study, FXYD5 was upregulated both at molecular and protein level in HGSOC patients with poor survival compared to those showing favourable outcome (*p* = 0.001 and *p* = 0.003, respectively) (Table 2 and Table 3).

**Table 2:** Clinical and pathological characteristics of 68 HGSOC patients and their association with FXYD5 mRNA expression evaluated by RT-qPCR

		FXYD5 mRNA		
Variable	Number of patients	Fold change	95% IC	*p*-value^a^
*Age at diagnosis*				
≥65 vs <65	36 vs 32	1.01	0.63–1.61	0.980
*Menopause*				
Post vs Pre	56 vs 12	1.02	0.55–1.89	0.956
*FIGO stage*				
III–IV vs I–II	64 vs 4	1.06	0.39–2.89	0.901
I–II–III vs IV	53 vs 15	1.16	0.66–2.06	0.588
IV vs III	15 vs 49	0.85	0.47–70.5	0.583
*Residual Tumour (cm)*				
RT > 0 vs RT = 0	52 vs 16	1.22	0.70–2.11	0.476
*Lymph nodal involvement*				
Pos vs Neg	16 vs 15	1.09	0.53–2.23	0.805
*Peritoneal cytology*				
Pos vs Neg	62 vs 5	1.14	0.46–2.81	0.766
*Platinum response*				
R vs S	39 vs 23	2.45	1.52–3.97	**<** **0.001**
R vs PS	39 vs 5	1.78	0.83–3.84	0.134
S vs PS	23 vs 5	0.73	0.42–1.26	0.239
*Relapse or Progression*				
Pos vs Neg	59 vs 9	2.01	1.03–3.94	**0.042**
*Overall Survival (months)*				
OS < 36 vs OS > 84	46 vs 22	2.27	1.43–3.60	**0.001**

*R* resistant, *S* sensitive, *PS* partially sensitive

Bold type has been used for statistically significant results

^a^two-tails *T* test

**Table 3:** Clinical and pathological characteristics of 48 HGSOC patients and their association with FXYD5 protein expression evaluated by immunohistochemistry

		FXYD5 protein expression				
Variable	No.	Score 1 + N° (%)	Score 2 + N° (%)	Score 3 + N° (%)		*p*-value^a^
*Age at diagnosis*						
≥65	29	14 (48)	10 (35)	5 (17)		0.585
<65	19	11 (58)	5 (26)	3 (16)		
*Menopause*						
Post	40	21 (53)	13 (32)	6 (15)		0.749
Pre	8	4 (50)	2 (25)	2 (25)		
*FIGO stage*						
I-II	4	3 (75)	1 (25)	0	I–II vs III–IV	0.294
III	32	15 (47)	11 (34)	6 (19)	I–II–III vs IV	0.704
IV	12	7 (58)	3 (25)	2 (17)	III vs IV	0.574
*Residual tumour (cm)*						
TR > 0	35	17 (49)	11 (31)	7 (20)		0.338
TR = 0	13	8 (61)	4 (31)	1 (8)		
*Lymph nodal involvement*						
Pos	12	6 (50)	3 (25)	3 (25)		0.511
Neg	14	8 (57)	5 (36)	1 (7)		
Unknown	22					
*Peritoneal cytology*						
Pos	42	21 (50)	13 (31)	8 (19)		0.182
Neg	5	3 (60)	2 (40)	0		
Unknown	1					
*Platinum response*						
Resistant	26	11 (42)	8 (31)	7 (27)	R vs S	**0.049**
Sensitive	19	13 (69)	5 (26)	1 (5)	R vs S + PS	**0.049**
Part Sens	2	1 (50)	1 (50)	0		
Unknown	1					
*Relapse/progression*						
Yes	41	19 (46)	14 (34)	8 (20)		**0.053**
No	7	6 (86)	1 (14)	0		
*Overall Survival (months)*						
OS < 36	30	11 (36.5)	11 (36.5)	8 (33)		**0.003**
OS > 84	18	14 (78)	4 (22)	0		

Bold type has been used for statistically significant results

^a^Mann–Whitney test

Moreover, the correlation between FXYD5 mRNA expression and clinical features revealed that the FXYD5 mean expression value was significantly higher in HGSOC patients showing platinum resistance compared to platinum sensitivity (FC = 2.45, 95% CI 1.52–3.97, *p* < 0.001), as well as in patients that experienced relapse or cancer progression compared to the others (FC = 2.01, 95% CI 1.03–3.94, *p* = 0.042) (Table 2).

Table 3 shows the correlations between FXYD5 protein expression and traditional clinicopathological factors in a subgroup of 48 patients. The percentage of tumours exhibiting FXYD5 strong positivity was significantly higher in platinum-resistant compared to platinum-sensitive cases (*p* = 0.049), and it was even more elevated in recurrent or progressive tumours compared to the others, even if with moderate statistical significance (*p* = 0.053) (Table 3). No other significant correlation was found.

### Evaluation of the prognostic potential of FXYD5 mRNA and FXYD5 protein expression

**Fig. 2:**
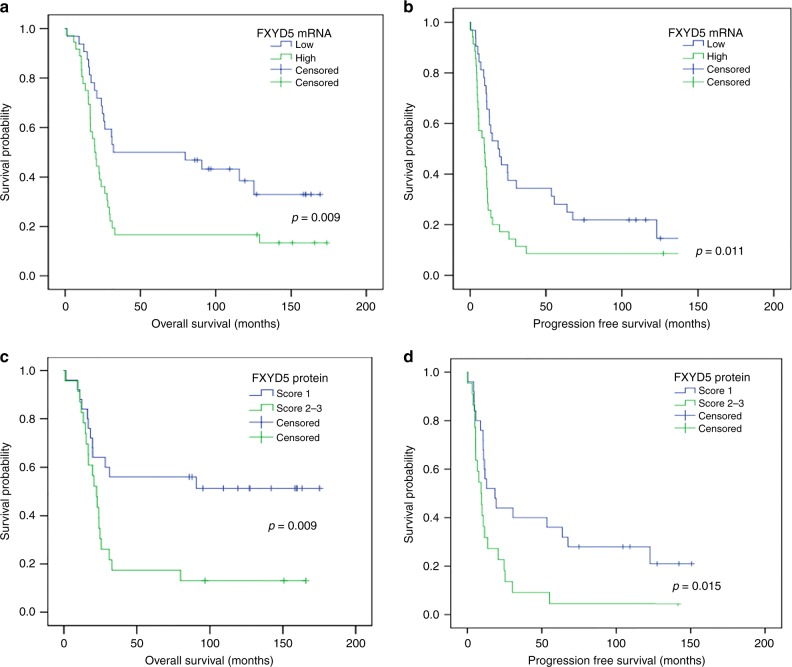
Kaplan–Meier overall survival (**a**, **c**) and progression free survival (**b**, **d**) curves for HGSOC patients according to FXYD5 mRNA and protein expression. The average log expression of FXYD5 mRNA was used as threshold to categorise patients (Low: ≤ 3.30; High: > 3.30). Kaplan–Meier plots showed a clear outcome difference between low and high FXYD5 expressing groups both at mRNA and protein level

In univariate survival analysis, OS and PFS were significantly shorter in patients with tumours exhibiting FXYD5 overexpression both at mRNA and protein level (Table 4 and Fig. 2).

**Table 4:** Univariate and multivariate survival analyses in relation to FXYD5 and clinicopathological parameters

	mRNA (high vs low)						Protein (score 2–3 vs score 1)					
	OS			PFS			OS			PFS		
Variables	HR	95% CI	*p*	HR	95% CI	*p*	HR	95% CI	*p*	HR	95% CI	p
*Univariate analysis*												
FXYD5	2.09	1.19–3.69	**0.011**	1.97	1.16–3.33	**0.012**	2.57	1.24–5.32	**0.011**	2.18	1.15–4.139	**0.017**
Age (years)												
≥65 vs <65	1.51	0.86–2.64	0.150	1.41	0.84–2.37	0.195	1.73	0.83–3.61	0.141	1.46	0.77–2.78	0.241
Menopausal status												
Post vs Pre	1.41	0.68–2.90	0.353	1.68	0.82–3.43	0.154	1.61	0.62–4.19	0.329	1.61	0.67–3.84	0.286
FIGO stage												
III and IV vs I and II	25.06	0–51–1221	0.104	3.93	0.95–16.23	**0.059**	25.89	0.31–21.87	0.151	3.81	0.91–15.99	0.067
Residual tumour (cm)												
RT > 0 vs RT = 0	2.72	1.27–5.84	**0.010**	1.76	0.95–3.28	0.074	2.84	1.09–7.41	**0.033**	1.40	0.70–2.81	0.344
Lymphnodal involvement												
Positive vs Negative	2.54	0.99–6.54	**0.053**	3.03	1.32–6.92	**0.009**	2.10	0.72–6.12	0.172	2.78	1.13–6.85	**0.026**
Peritoneal cytology												
Positive vs Negative	26.55	0.77–919.5	0.070	5.09	1.23–21.16	**0.025**	27.96	0.48–16.34	0.109	5.14	1.21–21.78	**0.026**
*Multivariate analysis*												
FXYD5	1.93	1.08–3.45	**0.025**	1.92	1.13–3.25	**0.016**	2.3	1.10–4.80	**0.026**	2.11	1.11–4.02	**0.023**
Age (years)	1.24	0.71–2.18	0.448	1.21	0.71–2.04	0.488	1.42	0.67–3.01	0.366	1.35	0.70–2.61	0.375
FIGO stage	0.74	0.45–1.21	0.226	0.99	0.63–1.55	0.951	0.88	0.48–1.60	0.665	1.05	0.64–1.71	0.852
Residual tumour	2.81	1.21–6.49	**0.016**	1.7	0.87–3.30	0.120	2.54	0.90–7.15	0.077	1.2	0.55–2.61	0.643

*HR* hazard ratio, *CI* confidence interval of the estimated HR

Bold type has been used for statistically significant results

After adjusting for age, residual tumour, and FIGO stage, multivariate cox proportional hazard model indicated that FXYD5 mRNA expression (HR = 1.93, *p* = 0.025), FXYD5 protein expression (HR = 2.30, *p* = 0.026) and residual tumour (HR = 2.81, *p* = 0.016) were independent prognostic factors for mortality, while only FXYD5, both at mRNA (HR = 1.92, *p* = 0.016) and protein level (HR = 2.11, *p* = 0.023), was an independent prognosticator in patients with disease recurrence/progression (Table 4).

## Discussion

In the present research, we aimed to identify and validate molecular markers distinguishing two extreme survival groups of HGSOC patients (OS < 3 years and OS > 7 years), commonly defined short-term and long-term survivors, respectively,^28^ characterised by similar initial clinical presentation, pathologic factors and treatments. Although pathological stage, histological grade and tumour histology constitute the most important prognostic factors in HGSOC and aid clinical decision-making process,^5^ their value in predicting recurrence and long-term prognosis in EOC patients remains still inadequate.^29^ Actually, limited data are available particularly for long-term HGSOC survivors, mainly because such patients, who survive 7–10 years after initial diagnosis, are rare and show a high prevalence of poor prognostic clinical factors at disease onset as like as short-term ones.^29^ Several gene expression studies have provided molecular profiles associated to extreme survivals in EOC,^28,30–34^ however a lack of concordance among them^29^ makes the identification of conserved prognostic factors still mandatory.

In the present study, we found 80 genes differentially expressed between long-term and short-term HGSOC survivors. Interestingly, pathway analyses showed that the ECM-receptor interaction pathway was significantly altered between the two extreme survival groups. Interactions between epithelial cells and the ECM are mediated by transmembrane molecules or cell-surface-associated components that lead to a direct or indirect control of cellular activities, such as adhesion, migration, differentiation, proliferation, and apoptosis modulating the hallmarks of cancer.^35^

Actually, among the differentially expressed genes, using three independent cohorts of patients, we successfully confirmed the altered expression of FXYD5, a single span type I membrane protein that plays multiple roles in regulation of cellular functions,^8^ both at mRNA and protein level. FXYD5 affects the functional properties of the Na^+^/K^+^ -ATPase activity that is implicated in many pathophysiological conditions, including cancer.^36^ Enhanced expression of FXYD5 reduced cell-cell adhesiveness in vitro, and enhanced the metastatic potential of liver, pancreatic and breast cancer cell lines in vivo.^7,37,38^ Moreover, the prognostic potential of FXYD5 has been investigated in several clinical studies and its overexpression has been significantly associated with poor outcome in different types of carcinoma.^7^

Regarding HGSOC, recently, a large-scale study performed by Raman et al. has described FXYD5 as a unique relevant survival-associated gene and driver for metastasis, through an in silico evaluation of gene expression and copy number amplifications from three independent public available datasets.^39^ In agreement with those results, in the current study, FXYD5 mRNA overexpression resulted an independent risk factor both for shorter OS and PFS. Moreover, the cBioPortal database analyses showed no FXYD5 point mutation but presence of copy number gain. Therefore, we supposed that its overexpression may be in part due to a copy number amplification, in agreement with previous studies.^39,40^

Our study showed an association between FXYD5 overexpression and carboplatin resistance in ovarian cancer. HGSOC may be intrinsically drug-resistant or develop resistance to chemotherapy during treatment and this represents a major obstacle to successful treatment. Several mechanisms of resistance to platinum-based chemotherapy have been proposed including, among others, alterations in transmembrane transport mechanisms, causing reduced intracellular cisplatin accumulation and enhanced epithelial-to-mesenchymal transition.^41^

A previous report has linked the aberrant expression of FXYD5 to in vitro drug resistance and apoptosis evasion in liver cancer stem cells.^37^ FXYD5 knockdown led to increased sensitivity to carboplatin, doxorubicin and fluorouracil and to reduced expression of the ABC transporter gene ABCG2 in hepatocellular carcinoma cells, suggesting a direct or indirect role of FXYD5 in chemotherapy resistance.^37^ Since the inhibition of the Na^+^/K^+^ - ATPase can also play a role in the downregulation of multiple drug-resistant proteins that allow cancer cells to resist to chemotherapy,^42^ and in particular the downregulation of Na^+^/K^+^ -ATPase beta subunit has been related to oxaliplatin resistance in ovarian cancer cell line,^43^ we suppose that one possible mechanisms linking FXYD5 to chemoresistance might involve the Na^+^/K^+^ -ATPase.

This is the first report describing FXYD5 among gynaecological tumours and their epithelial normal counterparts (healthy ovaries, fallopian tubes), both at transcript and protein expression level. Previously, other authors have investigated dysadherin expression in basal and parabasal cells of normal cervical epithelia and in squamous cell cervical carcinoma by immunohistochemistry and have correlated its upregulation to dismal prognosis.^44^

In the present study, we detected a negative immunostaining signal in all normal controls and a significant FXYD5 protein overexpression in tumour samples from short-term survivors compared to long-term ones. FXYD5 positive cells showed membranous, cytoplasmic or both type of staining in agreement with the expression pattern described for several types of carcinoma.^7^ Protein expression levels show a good correlation with transcript levels and its elevated expression score was significantly associated to platinum resistance, cancer progression/recurrence and worse prognosis. Our results are in agreement with other reports that documented a significant correlation between high dysadherin protein expression and enhanced tumoural invasiveness in breast cancer,^45^ increased metastatic potential and poor response to radiation therapy in head and neck cancer,^46^ and shorter survival time in non-small cell lung cancer.^47^

To date, in vitro and in vivo studies that explain the molecular mechanisms involving FXYD5 in the metastatic spread of ovarian malignancy are completely lacking. In accordance with other carcinomas,^7^ FXYD5 could confer to EOC cells a mesenchymal phenotype, modulate cellular junctions, influence chemokine production and migratory properties. This topic will be the object of our future research. Some studies have associated the pro-metastatic effect of FXYD5 to the transcriptional changes induced in E-cadherin^48^ or CCL2 chemokine and NF-kB,^49^ without demonstrating how FXYD5 actually alters the affected proteins described above.^8^ A recent investigation on molecular mechanisms linked to cancer progression in an in vivo breast cancer model pointed to the direct interaction between FXYD5 and its partner Na^+^/K^+^ -ATPase pump in multiple steps of the tumour spread process, such as epithelial-mesenchymal transition, loss of cell adhesion and gain of motility.^50^ In particular, FXYD5-dependent downregulation of the Na^+^/K^+^ -ATPase pump beta subunit (beta 1 isoform) mediated the metastatic progression of breast cancer in a mouse model^50^ while the suppression of the beta-subunit has been linked to the loss of tight junctions that promotes cell motility and cancer metastasis.^36^

In breast cancer, overexpression of FXYD5 was associated to increased activation of AKT.^38^ The inhibition of AKT suppressed FXYD5’s ability to activate NF-kB pathway and to promote cell mobility and tumour cell invasion.^38^ Further studies are necessary to identify pathways involving FXYD5, and tumours that could benefit of an FXYD5-targeted therapy, as suggested by a recent research reporting the efficacy of a novel antibody-drug conjugate for the selective growth inhibition of thyroid cancer cells expressing moderate to high dysadherin on cell surface.^51^

In conclusion, our study demonstrated that FXYD5 is an HGSOC-associated molecule, especially overexpressed in cases characterised by shorter survival, chemotherapy resistance and disease recurrence/progression, so it might be useful for identification of patients at higher risk of worse prognosis at the time of diagnosis.

## Supplementary information

Supplementary materials

## Data Availability

All data not included in this published article are available upon reasonable request.
