# Supplementary material for: FXYD5 (Dysadherin) upregulation predicts shorter survival and reveals platinum resistance in high-grade serous ovarian cancer patients
Source: Br J Cancer. 2019 Aug 22;121(7):584–92. doi: 10.1038/s41416-019-0553-z (PMC6889357; doi:10.1038/s41416-019-0553-z)

**SUPPLEMENTARY MATERIALS**

**FXYD5 (Dysadherin) upregulation predicts shorter survival and reveals platinum resistance in high-grade serous ovarian cancer patients**

##### Renata A Tassi^1^, Angela Gambino^2^, Laura Ardighieri^3^, Eliana Bignotti^1,4^, Paola Todeschini^1^, Chiara Romani^1,5^, Laura Zanotti^1^, Mattia Bugatti^3^, Fulvio Borella^6^, Dionyssios Katsaros^6^, Germana Tognon^4^, Enrico Sartori^2^, Franco Odicino^2^, Chiara Romualdi^7^*, Antonella Ravaggi^1^*

^1^ 'Angelo Nocivelli' Institute of Molecular Medicine, University of Brescia and ASST-Spedali Civili of Brescia, Brescia, Italy

^2^ Department of Clinical and Experimental Sciences, Division of Obstetrics and Gynecology University of Brescia, Brescia, Italy.

^3^ Department of Pathology, ASST Spedali Civili di Brescia, Brescia

^4^ Division of Obstetrics and Gynecology, ASST Spedali Civili di Brescia; Brescia, Italy.

^5^ Department of Molecular and Translational Medicine, University of Brescia; Brescia, Italy

^6^ Department of Surgical Sciences, Gynecologic Oncology, Città della Salute and S. Anna Hospital, University of Turin, Turin, Italy

^7^ Department of Biology, University of Padova, Padova, Italy.

* these authors contributed equally to this work

| **Supplementary Table S1…………………………….. page 3**  **Supplementary Table S2…………………………….. page 4**  **Supplementary Table S3…………………………….. page 6**  **Supplementary Figure S1…………………………… page 29**  **Supplementary Figure S2…………………………… page 30**  **Supplementary Figure S3…………………………… page 31**  **Table S1: Clinical and pathological characteristics of 68 HGSOC patients**   \| **Training set** \| **Validation set** \| \| **All** \| \| **P-value*** \| \| \| --- \| --- \| --- \| --- \| --- \| --- \| --- \| \| **Total number of patients** \| 39 \| \| \| 29 \| \| 68 \| \|  \| \| \| **Age (years)** \|  \| \| \|  \| \|  \| \| 0.451 \| \| \| Mean ± SD \| 65 ± 13 \| \| \| 63 ± 11 \| \| 64 ± 12 \| \|  \| \| \| Median (range) \| 67 (24-85) \| \| \| 63 (38-86) \| \| 66 (24-86) \| \|  \| \| \| **Menopausal status** \|  \| \| \|  \| \|  \| \| 0.472 \| \| \| Pre \| 8 (20.5%) \| \| \| 4 (13.8%) \| \| 12 (17.6%) \| \|  \| \| \| Post \| 31 (79.5%) \| \| \| 25 (86.2%) \| \| 56 (82.4%) \| \|  \| \| \| **FIGO stage** \|  \| \| \|  \| \|  \| \| 0.626 \| \| \| I \| 1 (2.6%) \| \| \| 0 \| \| 1 (1.4%) \| \|  \| \| \| II \| 2 (5.1%) \| \| \| 1 (3.5%) \| \| 3 (4.4%) \| \|  \| \| \| III \| 26 (66.7%) \| \| \| 23 (79.3%) \| \| 49 (72.1%) \| \|  \| \| \| IV \| 10 (25.6%) \| \| \| 5 (17.2%) \| \| 15 (22.1%) \| \|  \| \| \| **Residual Tumor (cm)** \|  \| \| \|  \| \|  \| \| 0.634 \| \| \| RT= 0 \| 10 (25.6%) \| \| \| 6 (20.7%) \| \| 16 (23.5%) \| \|  \| \| \| RT>0 \| 29 (74.4%) \| \| \| 23 (79.3%) \| \| 52 (76.5%) \| \|  \| \| \| **Peritoneal citology** \|  \| \| \|  \| \|  \| \| 0.377 \| \| \| Pos \| 34 (87.2%) \| \| \| 28 (96.6%) \| \| 62 (91.2%) \| \|  \| \| \| Neg \| 4 (10.3%) \| \| \| 1 (3.4%) \| \| 5 (7.4%) \| \|  \| \| \| Unknown \| 1 (2.5%) \| \| \| 0 \| \| 1 (1.4%) \| \|  \| \| \| **Lymph nodal involvement** \|  \| \| \|  \| \|  \| \| 0.062 \| \| \| Pos \| 9 (23.1%) \| \| \| 7 (24.1%) \| \| 16 (23.5%) \| \|  \| \| \| Neg \| 13 (33.3%) \| \| \| 2 (6.9%) \| \| 15 (22.1%) \| \|  \| \| \| Unknown \| 17 (43.6%) \| \| \| 20 (69.0%) \| \| 37 (54.4%) \| \|  \| \| \| **Relapse/Progression** \|  \| \| \|  \| \|  \| \| 0.401 \| \| \| Pos \| 35 (90%) \| \| \| 24 (82.8%) \| \| 59 (86.8%) \| \|  \| \| \| Neg \| 4 (10%) \| \| \| 5 (17.2%) \| \| 9 (13.2%) \| \|  \| \| \| **Platinum response** \|  \| \| \|  \| \|  \| \| 0.722 \| \| \| Sensitive \| 13 (33.3%) \| \| \| 10 (34.5%) \| \| 23 (33.8%) \| \|  \| \| \| Resistant \| 23 (59.0%) \| \| \| 16 (55.2%) \| \| 39 (57.4%) \| \|  \| \| \| Partially Sensitive \| 2 (5.1%) \| \| \| 3 (10.3%) \| \| 5 (7.4%) \| \|  \| \| \| Unknown \| 1 (2.6%) \| \| \| 0 \| \| 1 (1.4%) \| \|  \| \| \| **Vital status at last follow-up** \|  \| \| \|  \| \|  \| \| 0.887 \| \| \| Alive \| 8 (20.5%) \| \| \| 7 (24.1%) \| \| 15 (22.1%) \| \|  \| \| \| Dead of EOC \| 29 (74.4%) \| \| \| 22 (75.9%) \| \| 51 (75.0%) \| \|  \| \| \| Dead of other cause \| 2 (5.1%) \| \| \| 0 \| \| 2 (2.9%) \| \|  \| \| \| **Follow-up (months)** \|  \| \| \|  \| \|  \| \| 0.768 \| \| \| Mean ± SD \| 56 ± 59 \| \| \| 52 ± 50 \| \| 55 ± 55 \| \|  \| \| \| Median (range) \| 24 (1-170) \| \| \| 28 (1-174) \| \| 25 (1-174) \| \|  \| \|   In the comparison between training and validation set, P values were calculated with Pearson's chi- squared test of independence, and two tails t test (for age and follow-up)  **Table S2.** List of upregulated and downregulated genes in long-term survivors compared to short-term survivors. FDR < 0.1 | | | | | |
| --- | --- | --- | --- | --- | --- | --- | --- | --- | --- | --- | --- | --- | --- | --- | --- | --- | --- | --- | --- | --- | --- | --- | --- | --- | --- | --- | --- | --- | --- | --- | --- | --- | --- | --- | --- | --- | --- | --- | --- | --- | --- | --- | --- | --- | --- | --- | --- | --- | --- | --- | --- | --- | --- | --- | --- | --- | --- | --- | --- | --- | --- | --- | --- | --- | --- | --- | --- | --- | --- | --- | --- | --- | --- | --- | --- | --- | --- | --- | --- | --- | --- | --- | --- | --- | --- | --- | --- | --- | --- | --- | --- | --- | --- | --- | --- | --- | --- | --- | --- | --- | --- | --- | --- | --- | --- | --- | --- | --- | --- | --- | --- | --- | --- | --- | --- | --- | --- | --- | --- | --- | --- | --- | --- | --- | --- | --- | --- | --- | --- | --- | --- | --- | --- | --- | --- | --- | --- | --- | --- | --- | --- | --- | --- | --- | --- | --- | --- | --- | --- | --- | --- | --- | --- | --- | --- | --- | --- | --- | --- | --- | --- | --- | --- | --- | --- | --- | --- | --- | --- | --- | --- | --- | --- | --- | --- | --- | --- | --- | --- | --- | --- | --- | --- | --- | --- | --- | --- | --- | --- | --- | --- | --- | --- | --- | --- | --- | --- | --- | --- | --- | --- | --- | --- | --- | --- | --- | --- | --- | --- | --- | --- | --- | --- | --- | --- | --- | --- | --- | --- | --- | --- | --- | --- | --- | --- | --- | --- | --- | --- | --- | --- | --- | --- | --- | --- | --- | --- | --- | --- | --- | --- | --- | --- | --- | --- | --- | --- | --- | --- | --- | --- | --- | --- | --- | --- | --- | --- | --- | --- | --- | --- | --- | --- | --- | --- | --- | --- | --- | --- | --- | --- | --- | --- | --- | --- | --- | --- | --- | --- | --- | --- | --- | --- | --- | --- | --- | --- | --- | --- | --- | --- | --- | --- | --- | --- | --- | --- | --- | --- | --- | --- | --- | --- | --- | --- | --- | --- | --- | --- | --- | --- | --- | --- | --- | --- | --- | --- | --- | --- | --- | --- | --- | --- | --- | --- | --- | --- | --- | --- | --- | --- | --- | --- | --- | --- | --- | --- | --- | --- | --- | --- | --- | --- | --- | --- | --- | --- | --- | --- | --- | --- | --- | --- | --- | --- | --- | --- | --- | --- | --- | --- | --- | --- | --- | --- | --- | --- | --- | --- | --- | --- | --- | --- | --- | --- | --- | --- | --- | --- | --- | --- | --- | --- | --- | --- | --- | --- | --- | --- | --- | --- | --- |
| logFC: log fold change of the gene between long-OS and short-OS; P.Value: uncorrected p-value from the statistical test; adj.P.Value: FDR-corrected p-value. | | | | | |
| **Gene ID** | **Gene symbol** | **logFC** | **P.Value** | **adj.P.Val** |  |
| **81688** | **C6orf62** | **0.65278** | **6.69E-06** | **0.04431** |  |
| 9891 | NUAK1 | -1.17875 | 7.33E-06 | 0.04431 |  |
| **10384** | **BTN3A3** | **0.85477** | **1.48E-05** | **0.04907** |  |
| 57380 | MRS2 | 0.78799 | 2.06E-05 | 0.04907 |  |
| **6373** | **CXCL11** | **1.97411** | **2.89E-05** | **0.04907** |  |
| **64798** | **DEPTOR** | **1.48643** | **3.12E-05** | **0.04907** |  |
| 165 | AEBP1 | -1.32150 | 3.57E-05 | 0.04907 |  |
| 51567 | TDP2 | 0.66884 | 3.59E-05 | 0.04907 |  |
| 1307 | COL16A1 | -0.76090 | 4.15E-05 | 0.04907 |  |
| 54955 | C1orf109 | 0.51480 | 4.87E-05 | 0.04907 |  |
| 7389 | UROD | 0.53436 | 5.24E-05 | 0.04907 |  |
| 79717 | PPCS | 0.60063 | 6.32E-05 | 0.04907 |  |
| 1462 | VCAN | -1.73049 | 6.39E-05 | 0.04907 |  |
| 5159 | PDGFRB | -0.82403 | 6.60E-05 | 0.04907 |  |
| 10598 | AHSA1 | 0.63368 | 6.61E-05 | 0.04907 |  |
| 5118 | PCOLCE | -1.12644 | 6.68E-05 | 0.04907 |  |
| **10272** | **FSTL3** | **-0.52766** | **6.90E-05** | **0.04907** |  |
| 57535 | KIAA1324 | 0.92113 | 8.16E-05 | 0.04955 |  |
| 3292 | HSD17B1 | 0.61381 | 8.63E-05 | 0.04955 |  |
| 112464 | CAVIN3 | -0.69610 | 8.99E-05 | 0.04955 |  |
| **3093** | **UBE2K** | **0.55545** | **9.06E-05** | **0.04955** |  |
| **55107** | **ANO1** | **0.99061** | **9.71E-05** | **0.04955** |  |
| **53827** | **FXYD5** | **-1.39681** | **9.79E-05** | **0.04955** |  |
| 80194 | TMEM134 | 0.67023 | 9.83E-05 | 0.04955 |  |
| **65265** | **C8orf33** | **0.87642** | **0.00010** | **0.04988** |  |
| 7915 | ALDH5A1 | 0.70616 | 0.00012 | 0.05451 |  |
| 4718 | NDUFC2 | 0.48316 | 0.00013 | 0.05487 |  |
| 4017 | LOXL2 | -0.60888 | 0.00013 | 0.05487 |  |
| **2224** | **FDPS** | **0.54113** | **0.00014** | **0.05695** |  |
| 6903 | TBCC | 0.61573 | 0.00014 | 0.05750 |  |
| 5987 | TRIM27 | 0.64711 | 0.00015 | 0.05772 |  |
| 54802 | TRIT1 | 0.53141 | 0.00015 | 0.05772 |  |
| 2012 | EMP1 | -1.18758 | 0.00019 | 0.06841 |  |
| 3339 | HSPG2 | -0.51907 | 0.00019 | 0.06841 |  |
| 79084 | WDR77 | 0.50685 | 0.00020 | 0.06841 |  |
| 2766 | GMPR | 1.33896 | 0.00021 | 0.07181 |  |
| 26190 | FBXW2 | 0.41408 | 0.00022 | 0.07214 |  |
| 4116 | MAGOH | 0.58503 | 0.00023 | 0.07214 |  |
| 10969 | EBNA1BP2 | 0.61746 | 0.00025 | 0.07454 |  |
| 64963 | MRPS11 | 0.48941 | 0.00025 | 0.07454 |  |
| 160313 | KRT19P2 | -0.32838 | 0.00025 | 0.07454 |  |
| 23022 | PALLD | -0.91108 | 0.00027 | 0.07454 |  |
| 50626 | CYHR1 | 0.52120 | 0.00027 | 0.07454 |  |
| 5493 | PPL | -0.87623 | 0.00028 | 0.07454 |  |
| 7626 | ZNF75D | 0.41640 | 0.00029 | 0.07454 |  |
| 84617 | TUBB6 | -1.01458 | 0.00029 | 0.07454 |  |
| 26009 | ZZZ3 | 0.54701 | 0.00030 | 0.07454 |  |
| 1301 | COL11A1 | -2.54376 | 0.00031 | 0.07454 |  |
| 8346 | HIST1H2BI | 0.64049 | 0.00031 | 0.07454 |  |
| 11118 | BTN3A2 | 0.59303 | 0.00031 | 0.07454 |  |
| 55039 | TRMT12 | 0.66733 | 0.00031 | 0.07454 |  |
| 8642 | DCHS1 | -0.44966 | 0.00033 | 0.07590 |  |
| 7058 | THBS2 | -1.58772 | 0.00035 | 0.07806 |  |
| 9528 | TMEM59 | 0.49695 | 0.00035 | 0.07806 |  |
| 54107 | POLE3 | 0.72098 | 0.00038 | 0.08107 |  |
| 54345 | SOX18 | 1.49094 | 0.00039 | 0.08107 |  |
| 3675 | ITGA3 | -0.77779 | 0.00039 | 0.08107 |  |
| 54512 | EXOSC4 | 0.71413 | 0.00039 | 0.08107 |  |
| 29889 | GNL2 | 0.58426 | 0.00040 | 0.08158 |  |
| 54968 | TMEM70 | 0.36386 | 0.00043 | 0.08644 |  |
| 83468 | GLT8D2 | -0.99004 | 0.00044 | 0.08722 |  |
| 85236 | HIST1H2BK | 1.10388 | 0.00046 | 0.08820 |  |
| 28971 | AAMDC | 0.64666 | 0.00047 | 0.08820 |  |
| 28998 | MRPL13 | 0.89880 | 0.00047 | 0.08820 |  |
| 178 | AGL | 0.70247 | 0.00048 | 0.08820 |  |
| 27336 | HTATSF1 | 0.35980 | 0.00048 | 0.08820 |  |
| 6342 | SCP2 | 0.49276 | 0.00049 | 0.08855 |  |
| 9633 | TESMIN | 0.62897 | 0.00053 | 0.09405 |  |
| 6892 | TAPBP | 0.44997 | 0.00054 | 0.09405 |  |
| 3872 | KRT17 | -1.56961 | 0.00055 | 0.09433 |  |
| 9265 | CYTH3 | -0.28764 | 0.00056 | 0.09433 |  |
| 10450 | PPIE | 0.44244 | 0.00056 | 0.09433 |  |
| 28966 | SNX24 | -0.43810 | 0.00057 | 0.09485 |  |
| 10269 | ZMPSTE24 | 0.57995 | 0.00059 | 0.09495 |  |
| 65263 | PYCR3 | 0.58888 | 0.00059 | 0.09495 |  |
| 10630 | PDPN | -0.68837 | 0.00061 | 0.09685 |  |
| 34 | ACADM | 0.48685 | 0.00063 | 0.09879 |  |
| 7564 | ZNF16 | 0.52482 | 0.00065 | 0.09974 |  |
| 23764 | MAFF | -0.87673 | 0.00066 | 0.09974 |  |
| 11167 | FSTL1 | -0.83666 | 0.00067 | 0.09974 |  |
| 1948 | EFNB2 | -0.91680 | 0.00067 | 0.09974 |  |

| **Table S3. List of druggable genes among the 80 differentially expressed genes between long and short-term**  **survivors.** |
| --- |
| Actionable genes genes were derived from Drug Gene Interaction Database (doi: 10.1093/nar/gkx1143).   \| **search** \| **match** \| **match_type** \| **gene** \| **drug** \| **interaction** \| **sources** \| **pmids** \| \| --- \| --- \| --- \| --- \| --- \| --- \| --- \| --- \| \| 9891 \| NUAK1 \| Ambiguous \| NUAK1 \| Cerdulatinib \| inhibitor \| GuideToPharmacologyInteractions \|  \| \| 9891 \| NUAK1 \| Ambiguous \| NUAK1 \| CHEMBL384759 \| inhibitor \| GuideToPharmacologyInteractions \|  \| \| 9891 \| NUAK1 \| Ambiguous \| NUAK1 \| CHEMBL1229592 \| inhibitor \| GuideToPharmacologyInteractions \|  \| \| 64798 \| DEPTOR \| Definite \| DEPTOR \| AZD-8055 \| inhibitor \| MyCancerGenome \|  \| \| 64798 \| DEPTOR \| Definite \| DEPTOR \| OSI-027 \| inhibitor \| MyCancerGenome \|  \| \| 64798 \| DEPTOR \| Definite \| DEPTOR \| INK-128 \| inhibitor \| MyCancerGenome \|  \| \| 165 \| P2RY10 \| Ambiguous \| P2RY10 \| CHEMBL225155 \| agonist \| GuideToPharmacologyInteractions \|  \| \| 165 \| P2RY10 \| Ambiguous \| P2RY10 \| CHEMBL1368758 \| antagonist \| GuideToPharmacologyInteractions \|  \| \| 1307 \| PDE8A \| Ambiguous \| PDE8A \| DIPYRIDAMOLE \| inhibitor \| GuideToPharmacologyInteractions\|ChemblInteractions \|  \| \| 1307 \| PDE8A \| Ambiguous \| PDE8A \| FLAVOXATE HYDROCHLORIDE \| inhibitor \| ChemblInteractions \|  \| \| 1307 \| PDE8A \| Ambiguous \| PDE8A \| PENTOXIFYLLINE \| inhibitor \| ChemblInteractions \|  \| \| 1307 \| PDE8A \| Ambiguous \| PDE8A \| ISOBUTYLMETHYLXANTHINE \|  \| DrugBank \| 10592235 \| \| 7389 \| UROD \| Definite \| UROD \| COPROPORPHYRINOGEN-III \|  \| DrugBank \| 17139284\|17016423 \| \| 1462 \| VCAN \| Ambiguous \| VCAN \| HYALURONIC ACID \| binder \| DrugBank \| 2466833\|2469524 \| \| 1462 \| VCAN \| Ambiguous \| VCAN \| CYCLOSPORINE \|  \| NCI \| 14974815 \| \| 1462 \| CAMK2D \| Ambiguous \| CAMK2D \| CHEMBL605003 \|  \| DrugBank \| 10592235 \| \| 5159 \| PDGFRB \| Definite \| PDGFRB \| SORAFENIB \| antagonist\|inhibitor \| TALC\|MyCancerGenome\|TdgClinicalTrial\|ClearityFoundationClinicalTrial\|TEND\|DrugBank\|MyCancerGenomeClinicalTrial \| 16425993\|17545544\|17619763 \| \| 5159 \| PDGFRB \| Definite \| PDGFRB \| DASATINIB \| antagonist\|inhibitor \| TdgClinicalTrial\|ChemblInteractions\|TEND\|DrugBank\|MyCancerGenomeClinicalTrial \| 20072833\|16497876\|16436588 \| \| 5159 \| PDGFRB \| Definite \| PDGFRB \| SUNITINIB \| inhibitor \| TALC\|MyCancerGenome\|TdgClinicalTrial\|GuideToPharmacologyInteractions\|TEND\|DrugBank \| 16425993\|12538485\|14753710\|12748309\|15557593 \| \| 5159 \| PDGFRB \| Definite \| PDGFRB \| PAZOPANIB \| inhibitor \| TALC\|MyCancerGenome\|GuideToPharmacologyInteractions\|DrugBank\|MyCancerGenomeClinicalTrial \| 17288876 \| \| 5159 \| PDGFRB \| Definite \| PDGFRB \| REGORAFENIB \| inhibitor \| TALC\|MyCancerGenome\|ChemblInteractions\|DrugBank\|MyCancerGenomeClinicalTrial \|  \| \| 5159 \| PDGFRB \| Definite \| PDGFRB \| CEDIRANIB \| inhibitor \| TALC\|GuideToPharmacologyInteractions\|ChemblInteractions \|  \| \| 5159 \| PDGFRB \| Definite \| PDGFRB \| PHENOBARBITAL \| inhibitor \| GuideToPharmacologyInteractions \|  \| \| 5159 \| PDGFRB \| Definite \| PDGFRB \| CRENOLANIB \| inhibitor \| GuideToPharmacologyInteractions\|ChemblInteractions \|  \| \| 5159 \| PDGFRB \| Definite \| PDGFRB \| DOVITINIB \| inhibitor \| TdgClinicalTrial\|ClearityFoundationClinicalTrial\|GuideToPharmacologyInteractions\|ChemblInteractions \|  \| \| 5159 \| PDGFRB \| Definite \| PDGFRB \| Famitinib \| inhibitor \| GuideToPharmacologyInteractions\|ChemblInteractions \|  \| \| 5159 \| PDGFRB \| Definite \| PDGFRB \| CHEMBL377193 \| inhibitor \| GuideToPharmacologyInteractions \|  \| \| 5159 \| PDGFRB \| Definite \| PDGFRB \| CHEMBL406375 \| inhibitor \| GuideToPharmacologyInteractions \|  \| \| 5159 \| PDGFRB \| Definite \| PDGFRB \| CHEMBL1908396 \| inhibitor \| GuideToPharmacologyInteractions \|  \| \| 5159 \| PDGFRB \| Definite \| PDGFRB \| LINIFANIB \| inhibitor \| MyCancerGenome\|TdgClinicalTrial\|GuideToPharmacologyInteractions\|ChemblInteractions \|  \| \| 5159 \| PDGFRB \| Definite \| PDGFRB \| LUCITANIB \| inhibitor \| GuideToPharmacologyInteractions \|  \| \| 5159 \| PDGFRB \| Definite \| PDGFRB \| MASITINIB \| inhibitor \| TdgClinicalTrial\|GuideToPharmacologyInteractions\|ChemblInteractions\|MyCancerGenomeClinicalTrial \|  \| \| 5159 \| PDGFRB \| Definite \| PDGFRB \| MK-2461 \| inhibitor \| GuideToPharmacologyInteractions \|  \| \| 5159 \| PDGFRB \| Definite \| PDGFRB \| NINTEDANIB \| inhibitor \| TALC\|MyCancerGenome\|TdgClinicalTrial\|ClearityFoundationClinicalTrial\|GuideToPharmacologyInteractions \|  \| \| 5159 \| PDGFRB \| Definite \| PDGFRB \| ORANTINIB \| inhibitor \| MyCancerGenome\|GuideToPharmacologyInteractions\|ChemblInteractions \|  \| \| 5159 \| PDGFRB \| Definite \| PDGFRB \| CHEMBL120077 \| inhibitor \| GuideToPharmacologyInteractions \|  \| \| 5159 \| PDGFRB \| Definite \| PDGFRB \| QUIZARTINIB \| inhibitor \| GuideToPharmacologyInteractions\|ChemblInteractions \|  \| \| 5159 \| PDGFRB \| Definite \| PDGFRB \| SEMAXANIB \| inhibitor \| GuideToPharmacologyInteractions \|  \| \| 5159 \| PDGFRB \| Definite \| PDGFRB \| SU-11652 \| inhibitor \| GuideToPharmacologyInteractions \|  \| \| 5159 \| PDGFRB \| Definite \| PDGFRB \| SU-14813 \| inhibitor \| GuideToPharmacologyInteractions\|ChemblInteractions \|  \| \| 5159 \| PDGFRB \| Definite \| PDGFRB \| TANDUTINIB \| inhibitor \| MyCancerGenome\|GuideToPharmacologyInteractions\|ChemblInteractions \|  \| \| 5159 \| PDGFRB \| Definite \| PDGFRB \| VATALANIB \| inhibitor \| TALC\|TdgClinicalTrial\|GuideToPharmacologyInteractions\|ChemblInteractions \|  \| \| 5159 \| PDGFRB \| Definite \| PDGFRB \| TELATINIB \| inhibitor \| TALC\|MyCancerGenome\|TdgClinicalTrial\|ChemblInteractions \|  \| \| 5159 \| PDGFRB \| Definite \| PDGFRB \| MOTESANIB \| inhibitor \| TALC\|MyCancerGenome\|TdgClinicalTrial\|ChemblInteractions \|  \| \| 5159 \| PDGFRB \| Definite \| PDGFRB \| AXITINIB \| inhibitor \| TALC\|MyCancerGenome\|TdgClinicalTrial \|  \| \| 5159 \| PDGFRB \| Definite \| PDGFRB \| IMATINIB \| inhibitor \| MyCancerGenome\|TdgClinicalTrial\|OncoKB\|CGI\|CIViC\|TEND\|FDA \| 1516603\|12676775\|23835704\|22897847\|24687085\|21128251\|12181402\|15746584\|15503291\|19620561\|18950453\|24963404\|16960151\|15681532\|14504092\|20439456 \| \| 5159 \| PDGFRB \| Definite \| PDGFRB \| X-82 \| inhibitor \| MyCancerGenome\|ChemblInteractions \|  \| \| 5159 \| PDGFRB \| Definite \| PDGFRB \| NILOTINIB \| inhibitor \| MyCancerGenomeClinicalTrial \|  \| \| 5159 \| PDGFRB \| Definite \| PDGFRB \| XL-820 \| inhibitor \| TALC\|MyCancerGenome\|TdgClinicalTrial\|ChemblInteractions\|DrugBank \|  \| \| 5159 \| PDGFRB \| Definite \| PDGFRB \| SORAFENIB TOSYLATE \| inhibitor \| ChemblInteractions\|MyCancerGenomeClinicalTrial \|  \| \| 5159 \| PDGFRB \| Definite \| PDGFRB \| SUNITINIB MALATE \| inhibitor \| ChemblInteractions \|  \| \| 5159 \| PDGFRB \| Definite \| PDGFRB \| ENMD-981693 \| inhibitor \| ChemblInteractions \|  \| \| 5159 \| PDGFRB \| Definite \| PDGFRB \| TAK-593 \| inhibitor \| ChemblInteractions \|  \| \| 5159 \| PDGFRB \| Definite \| PDGFRB \| RG-1530 \| inhibitor \| ChemblInteractions \|  \| \| 5159 \| PDGFRB \| Definite \| PDGFRB \| Puquitinib \| inhibitor \| ChemblInteractions \|  \| \| 5159 \| PDGFRB \| Definite \| PDGFRB \| XL-999 \| inhibitor \| TdgClinicalTrial\|ChemblInteractions\|DrugBank \|  \| \| 5159 \| PDGFRB \| Definite \| PDGFRB \| MIDOSTAURIN \| inhibitor \| ChemblInteractions \|  \| \| 5159 \| PDGFRB \| Definite \| PDGFRB \| CDP-860 \| inhibitor \| ChemblInteractions \|  \| \| 5159 \| PDGFRB \| Definite \| PDGFRB \| BECAPLERMIN \| agonist \| TdgClinicalTrial\|ChemblInteractions\|TEND\|DrugBank \| 18686746 \| \| 5159 \| PDGFRB \| Definite \| PDGFRB \| PAZOPANIB HYDROCHLORIDE \| inhibitor \| ChemblInteractions \|  \| \| 5159 \| PDGFRB \| Definite \| PDGFRB \| FORETINIB \| inhibitor \| ChemblInteractions \|  \| \| 5159 \| PDGFRB \| Definite \| PDGFRB \| CEP-2563 \| inhibitor \| ChemblInteractions \|  \| \| 5159 \| PDGFRB \| Definite \| PDGFRB \| SU-014813 \| inhibitor \| ChemblInteractions \|  \| \| 5159 \| PDGFRB \| Definite \| PDGFRB \| JI-101 \| inhibitor \| ChemblInteractions \|  \| \| 5159 \| PDGFRB \| Definite \| PDGFRB \| ILORASERTIB \| inhibitor \| ChemblInteractions \|  \| \| 5159 \| PDGFRB \| Definite \| PDGFRB \| PD-0166285 (CHEMBL3545196) \| inhibitor \| ChemblInteractions \|  \| \| 5159 \| PDGFRB \| Definite \| PDGFRB \| IMATINIB MESYLATE \| inhibitor \| ChemblInteractions \|  \| \| 5159 \| PDGFRB \| Definite \| PDGFRB \| NINTEDANIB ESYLATE \| inhibitor \| ChemblInteractions \|  \| \| 5159 \| PDGFRB \| Definite \| PDGFRB \| CM-082 \| inhibitor \| ChemblInteractions \|  \| \| 5159 \| PDGFRB \| Definite \| PDGFRB \| Anlotinib \| inhibitor \| ChemblInteractions \|  \| \| 5159 \| PDGFRB \| Definite \| PDGFRB \| TG100-801 \| inhibitor \| ChemblInteractions \|  \| \| 5159 \| PDGFRB \| Definite \| PDGFRB \| PEGPLERANIB SODIUM \|  \| TdgClinicalTrial \|  \| \| 5159 \| PDGFRB \| Definite \| PDGFRB \| LENVATINIB \|  \| TdgClinicalTrial \|  \| \| 5159 \| PDGFRB \| Definite \| PDGFRB \| AMUVATINIB \|  \| TdgClinicalTrial \|  \| \| 3292 \| HSD17B1 \| Ambiguous \| HSD17B1 \| ANDROSTENEDIONE \| inducer \| DrugBank \| 17139284\|17016423 \| \| 3292 \| HSD17B1 \| Ambiguous \| HSD17B1 \| STANOLONE \|  \| DrugBank \|  \| \| 3292 \| HSD17B1 \| Ambiguous \| HSD17B1 \| CHEMBL1161862 \|  \| DrugBank \| 17139284\|17016423 \| \| 3292 \| HSD17B1 \| Ambiguous \| HSD17B1 \| EQUILIN \|  \| DrugBank \| 10592235\|17139284\|17016423 \| \| 3292 \| HSD17B1 \| Ambiguous \| HSD17B1 \| CHEMBL1230438 \|  \| DrugBank \| 10592235\|17139284\|17016423 \| \| 3292 \| HSD17B1 \| Ambiguous \| HSD17B1 \| CHEMBL371948 \|  \| DrugBank \| 17139284\|17016423 \| \| 3292 \| HSD17B1 \| Ambiguous \| HSD17B1 \| CHEMBL1161866 \|  \| DrugBank \| 3463506\|3164265\|17139284\|2615366\|17016423 \| \| 3292 \| HSD17B1 \| Ambiguous \| HSD17B1 \| PRASTERONE \|  \| DrugBank \| 10592235\|17139284\|17016423 \| \| 55107 \| ANO1 \| Definite \| ANO1 \| cA2 \| activator \| GuideToPharmacologyInteractions \|  \| \| 55107 \| ANO1 \| Definite \| ANO1 \| ANTHRACENE-9-CARBOXYLIC ACID \| channel blocker \| GuideToPharmacologyInteractions \|  \| \| 55107 \| ANO1 \| Definite \| ANO1 \| METRONIDAZOLE \| channel blocker \| GuideToPharmacologyInteractions \|  \| \| 55107 \| ANO1 \| Definite \| ANO1 \| FLUFENAMIC ACID \| channel blocker \| GuideToPharmacologyInteractions \|  \| \| 55107 \| ANO1 \| Definite \| ANO1 \| FLUOXETINE \| channel blocker \| GuideToPharmacologyInteractions \|  \| \| 55107 \| ANO1 \| Definite \| ANO1 \| CHEMBL23050 \| channel blocker \| GuideToPharmacologyInteractions \|  \| \| 55107 \| ANO1 \| Definite \| ANO1 \| MIBEFRADIL \| channel blocker \| GuideToPharmacologyInteractions \|  \| \| 55107 \| ANO1 \| Definite \| ANO1 \| NIFLUMIC ACID \| channel blocker \| GuideToPharmacologyInteractions \|  \| \| 55107 \| ANO1 \| Definite \| ANO1 \| 5-NITRO-2-PHENYLPROPYLAMINOBENZOIC ACID [NPPB] \| channel blocker \| GuideToPharmacologyInteractions \|  \| \| 55107 \| ANO1 \| Definite \| ANO1 \| CHEMBL1162149 \| channel blocker \| GuideToPharmacologyInteractions \|  \| \| 55107 \| ANO1 \| Definite \| ANO1 \| TANNIC ACID \| channel blocker \| GuideToPharmacologyInteractions \|  \| \| 55107 \| ANO1 \| Definite \| ANO1 \| CROFELEMER \| inhibitor\|antagonist\|blocker \| GuideToPharmacologyInteractions\|ChemblInteractions\|DrugBank \| 19808995 \| \| 7915 \| ALDH5A1 \| Ambiguous \| ALDH5A1 \| SUCCINIC ACID \| inhibitor \| DrugBank\|TTD \| 16689938\|16942602\|17584458\|17619587\|15853764\|11752352 \| \| 7915 \| ALDH5A1 \| Ambiguous \| ALDH5A1 \| CHLORMERODRIN \| inhibitor \| DrugBank \| 5692397\|11752352\|17139284\|17016423 \| \| 7915 \| ALDH5A1 \| Ambiguous \| ALDH5A1 \| DIVALPROEX SODIUM \| inhibitor \| ChemblInteractions \|  \| \| 7915 \| ALDH5A1 \| Ambiguous \| ALDH5A1 \| VALPROATE SODIUM \| inhibitor \| ChemblInteractions \|  \| \| 7915 \| ALDH5A1 \| Ambiguous \| ALDH5A1 \| VALPROIC ACID \| inhibitor \| ChemblInteractions \|  \| \| 7915 \| ALDH5A1 \| Ambiguous \| ALDH5A1 \| CHEMBL378577 \| inhibitor \| GuideToPharmacologyInteractions \|  \| \| 7915 \| ALDH5A1 \| Ambiguous \| ALDH5A1 \| CHEMBL1161866 \|  \| DrugBank \| 17139284\|17016423 \| \| 4718 \| NDUFC2 \| Ambiguous \| NDUFC2 \| CARVEDILOL \| inhibitor \| DrugBank \| 10666308 \| \| 4718 \| NDUFC2 \| Ambiguous \| NDUFC2 \| ME-344 \| inhibitor \| ChemblInteractions \|  \| \| 4718 \| NDUFC2 \| Ambiguous \| NDUFC2 \| NV-128 \| inhibitor \| ChemblInteractions \|  \| \| 4718 \| NDUFC2 \| Ambiguous \| NDUFC2 \| METFORMIN HYDROCHLORIDE \| inhibitor \| ChemblInteractions \|  \| \| 4718 \| NDUFC2 \| Ambiguous \| NDUFC2 \| CHEMBL1161866 \|  \| DrugBank \| 16898010\|17015645\|17496098\|17513495\|17614984 \| \| 4017 \| LOXL2 \| Ambiguous \| LOXL2 \| Simtuzumab \| inhibitor \| GuideToPharmacologyInteractions\|ChemblInteractions \|  \| \| 2224 \| FDPS \| Ambiguous \| FDPS \| ZOLEDRONIC ACID \| inhibitor \| TdgClinicalTrial\|GuideToPharmacologyInteractions\|ChemblInteractions\|TEND\|DrugBank \| 11160603\|17477829\|11752352\|17535895 \| \| 2224 \| FDPS \| Ambiguous \| FDPS \| ALENDRONIC ACID \| inhibitor \| TdgClinicalTrial\|GuideToPharmacologyInteractions\|TEND \|  \| \| 2224 \| FDPS \| Ambiguous \| FDPS \| (CYCLOHEXYLAMINO)METHYLENEDIPHOSPHONIC ACID \| inhibitor \| GuideToPharmacologyInteractions \|  \| \| 2224 \| FDPS \| Ambiguous \| FDPS \| DOXAPRAM \| inhibitor \| GuideToPharmacologyInteractions \|  \| \| 2224 \| FDPS \| Ambiguous \| FDPS \| CHEMBL437758 \| inhibitor \| GuideToPharmacologyInteractions \|  \| \| 2224 \| FDPS \| Ambiguous \| FDPS \| IBANDRONIC ACID \| inhibitor \| TdgClinicalTrial\|GuideToPharmacologyInteractions\|TEND \|  \| \| 2224 \| FDPS \| Ambiguous \| FDPS \| MINODRONIC ACID \| inhibitor \| TdgClinicalTrial\|GuideToPharmacologyInteractions \|  \| \| 2224 \| FDPS \| Ambiguous \| FDPS \| CHEMBL99553 \| inhibitor \| GuideToPharmacologyInteractions \|  \| \| 2224 \| FDPS \| Ambiguous \| FDPS \| CHEMBL294192 \| inhibitor \| GuideToPharmacologyInteractions \|  \| \| 2224 \| FDPS \| Ambiguous \| FDPS \| DROMOSTANOLONE PROPIONATE \| inhibitor \| GuideToPharmacologyInteractions \|  \| \| 2224 \| FDPS \| Ambiguous \| FDPS \| DROPERIDOL \| inhibitor \| GuideToPharmacologyInteractions \|  \| \| 2224 \| FDPS \| Ambiguous \| FDPS \| DORZOLAMIDE \| inhibitor \| GuideToPharmacologyInteractions \|  \| \| 2224 \| FDPS \| Ambiguous \| FDPS \| PAMIDRONIC ACID \| inhibitor \| TdgClinicalTrial\|GuideToPharmacologyInteractions\|TEND \|  \| \| 2224 \| FDPS \| Ambiguous \| FDPS \| PIRIDRONIC ACID \| inhibitor \| GuideToPharmacologyInteractions \|  \| \| 2224 \| FDPS \| Ambiguous \| FDPS \| RISEDRONIC ACID \| inhibitor \| TdgClinicalTrial\|GuideToPharmacologyInteractions\|TEND \|  \| \| 2224 \| FDPS \| Ambiguous \| FDPS \| ALENDRONATE SODIUM \| inhibitor \| ChemblInteractions \|  \| \| 2224 \| FDPS \| Ambiguous \| FDPS \| RISEDRONATE SODIUM \| inhibitor \| ChemblInteractions \|  \| \| 2224 \| FDPS \| Ambiguous \| FDPS \| IBANDRONATE SODIUM \| inhibitor \| ChemblInteractions \|  \| \| 2224 \| FDPS \| Ambiguous \| FDPS \| PAMIDRONATE DISODIUM \| inhibitor \| ChemblInteractions \|  \| \| 2224 \| FDPS \| Ambiguous \| FDPS \| GERANYL DIPHOSPHATE \|  \| DrugBank \| 10592235 \| \| 2224 \| FDPS \| Ambiguous \| FDPS \| DIMETHYLALLYL DIPHOSPHATE \|  \| DrugBank \| 10592235 \| \| 2224 \| FDPS \| Ambiguous \| FDPS \| MEDRONIC ACID \|  \| PharmGKB \|  \| \| 2224 \| FDPS \| Ambiguous \| FDPS \| CHEMBL1229266 \|  \| DrugBank \| 10592235 \| \| 2224 \| FDPS \| Ambiguous \| FDPS \| FARNESYL DIPHOSPHATE \|  \| DrugBank \| 10592235 \| \| 2224 \| FDPS \| Ambiguous \| FDPS \| ISOPENTENYLPYROPHOSPHATE \|  \| DrugBank \| 10592235 \| \| 6903 \| MAT1A \| Ambiguous \| MAT1A \| S-ADENOSYL-L-METHIONINE \| cofactor \| DrugBank \| 12660248\|12631701\|11301045\|12671891\|12060674 \| \| 6903 \| MAT1A \| Ambiguous \| MAT1A \| CHEMBL295971 \|  \| DrugBank \| 10592235\|17139284\|17016423 \| \| 54802 \| TRIT1 \| Definite \| TRIT1 \| AZACITIDINE \|  \| NCI \| 2479825 \| \| 2012 \| EMP1 \| Ambiguous \| EMP1 \| GEFITINIB \|  \| NCI \| 16087880 \| \| 3339 \| HSPG2 \| Ambiguous \| HSPG2 \| PALIFERMIN \|  \| TdgClinicalTrial\|DrugBank \| 16989989\|10593896\|9788974\|14753849 \| \| 3339 \| HSPG2 \| Ambiguous \| HSPG2 \| CYCLOSPORINE \|  \| NCI \| 14974815 \| \| 2766 \| GMPR \| Ambiguous \| GMPR \| CHEMBL283807 \|  \| DrugBank \| 17139284\|17016423 \| \| 2766 \| CD38 \| Ambiguous \| CD38 \| DARATUMUMAB \| antibody\| inhibitor \| MyCancerGenome\|GuideToPharmacologyInteractions\|ChemblInteractions \|  \| \| 2766 \| CD38 \| Ambiguous \| CD38 \| ISATUXIMAB \| antibody \| GuideToPharmacologyInteractions \|  \| \| 2766 \| CD38 \| Ambiguous \| CD38 \| SAR-650984 \|  \| TdgClinicalTrial \|  \| \| 2766 \| CD38 \| Ambiguous \| CD38 \| HuMax-CD38 \|  \| TdgClinicalTrial \|  \| \| 2766 \| CD38 \| Ambiguous \| CD38 \| THROMBIN \|  \| NCI \| 9654134 \| \| 4116 \| GALE \| Ambiguous \| GALE \| URIDINE DIPHOSPHATE GLUCOSE \|  \| DrugBank \| 10592235\|17139284\|17016423 \| \| 4116 \| GALE \| Ambiguous \| GALE \| TETRAMETHYL AMMONIUM ION \|  \| DrugBank \| 17139284\|17016423 \| \| 4116 \| GALE \| Ambiguous \| GALE \| CHEMBL439009 \|  \| DrugBank \| 11279193 \| \| 4116 \| GALE \| Ambiguous \| GALE \| CHEMBL50267 \|  \| DrugBank \| 17139284\|17016423 \| \| 10969 \| SLC22A5 \| Ambiguous \| SLC22A5 \| LEVOCARNITINE \|  \| DrugBank \| 12644265\|12175785\|12181285\|12183691\|12635840 \| \| 84617 \| TUBB6 \| Definite \| TUBB6 \| INDIBULIN \| inhibitor \| ChemblInteractions \|  \| \| 84617 \| TUBB6 \| Definite \| TUBB6 \| CABAZITAXEL \| inhibitor \| ChemblInteractions \|  \| \| 84617 \| TUBB6 \| Definite \| TUBB6 \| CROLIBULIN \| inhibitor \| ChemblInteractions \|  \| \| 84617 \| TUBB6 \| Definite \| TUBB6 \| VINFLUNINE \| inhibitor \| ChemblInteractions \|  \| \| 84617 \| TUBB6 \| Definite \| TUBB6 \| FOSBRETABULIN DISODIUM \| inhibitor \| ChemblInteractions \|  \| \| 84617 \| TUBB6 \| Definite \| TUBB6 \| PLINABULIN \| inhibitor \| ChemblInteractions \|  \| \| 84617 \| TUBB6 \| Definite \| TUBB6 \| TRASTUZUMAB EMTANSINE \| inhibitor \| ChemblInteractions \|  \| \| 84617 \| TUBB6 \| Definite \| TUBB6 \| BRENTUXIMAB VEDOTIN \| inhibitor \| ChemblInteractions \|  \| \| 84617 \| TUBB6 \| Definite \| TUBB6 \| PACLITAXEL \| inhibitor \| ChemblInteractions \|  \| \| 84617 \| TUBB6 \| Definite \| TUBB6 \| DOCETAXEL HYDRATE \| inhibitor \| ChemblInteractions \|  \| \| 84617 \| TUBB6 \| Definite \| TUBB6 \| ERIBULIN MESYLATE \| inhibitor \| ChemblInteractions \|  \| \| 84617 \| TUBB6 \| Definite \| TUBB6 \| COLCHICINE \| inhibitor \| ChemblInteractions \|  \| \| 84617 \| TUBB6 \| Definite \| TUBB6 \| IXABEPILONE \| inhibitor \| ChemblInteractions \|  \| \| 84617 \| TUBB6 \| Definite \| TUBB6 \| VINCRISTINE SULFATE \| inhibitor \| ChemblInteractions \|  \| \| 84617 \| TUBB6 \| Definite \| TUBB6 \| VINBLASTINE SULFATE \| inhibitor \| ChemblInteractions \|  \| \| 84617 \| TUBB6 \| Definite \| TUBB6 \| VINORELBIN DITARTRATE \| inhibitor \| ChemblInteractions \|  \| \| 84617 \| TUBB6 \| Definite \| TUBB6 \| LEXIBULIN \| inhibitor \| ChemblInteractions\|DrugBank \|  \| \| 84617 \| TUBB6 \| Definite \| TUBB6 \| FOSBRETABULIN TROMETHAMINE \|  \| ChemblInteractions \|  \| \| 84617 \| TUBB6 \| Definite \| TUBB6 \| SAGOPILONE \|  \| ChemblInteractions \|  \| \| 84617 \| TUBB6 \| Definite \| TUBB6 \| PACLITAXEL POLIGLUMEX \|  \| ChemblInteractions \|  \| \| 84617 \| TUBB6 \| Definite \| TUBB6 \| VERUBULIN \|  \| ChemblInteractions \|  \| \| 84617 \| TUBB6 \| Definite \| TUBB6 \| DAVUNETIDE \|  \| ChemblInteractions \|  \| \| 1301 \| COL11A1 \| Ambiguous \| COL11A1 \| OCRIPLASMIN \|  \| ChemblInteractions \|  \| \| 1301 \| COL11A1 \| Ambiguous \| COL11A1 \| COLLAGENASE CLOSTRIDIUM HISTOLYTICUM \|  \| ChemblInteractions \|  \| \| 1301 \| PDE4B \| Ambiguous \| PDE4B \| ADENOSINE PHOSPHATE \| product of \| DrugBank \| 16843095\|17333137\|17088426 \| \| 1301 \| PDE4B \| Ambiguous \| PDE4B \| THEOPHYLLINE \| inhibitor \| TdgClinicalTrial\|ChemblInteractions\|TEND\|DrugBank \| 15639300 \| \| 1301 \| PDE4B \| Ambiguous \| PDE4B \| DYPHYLLINE \| inhibitor \| TdgClinicalTrial\|ChemblInteractions\|TEND\|DrugBank \| 7925603\|17139284\|17016423\|225216 \| \| 1301 \| PDE4B \| Ambiguous \| PDE4B \| PENTOXIFYLLINE \| inhibitor \| TdgClinicalTrial\|ChemblInteractions\|TEND\|DrugBank \| 17139284\|17016423 \| \| 1301 \| PDE4B \| Ambiguous \| PDE4B \| ENPROFYLLINE \| inhibitor \| TdgClinicalTrial\|TEND\|DrugBank \| 17139284\|17016423 \| \| 1301 \| PDE4B \| Ambiguous \| PDE4B \| ILOPROST \| inducer \| DrugBank \| 12952271\|11551870\|12441759\|2461561 \| \| 1301 \| PDE4B \| Ambiguous \| PDE4B \| PAPAVERINE \| inhibitor \| TdgClinicalTrial\| TEND\|DrugBank \| 19231363\|12646997\|17139284\|17016423 \| \| 1301 \| PDE4B \| Ambiguous \| PDE4B \| THEOBROMINE \| inhibitor \| DrugBank \| 18568240\|11692087\|15095008\|17514358 \| \| 1301 \| PDE4B \| Ambiguous \| PDE4B \| INAMRINONE \| inhibitor \| TdgClinicalTrial\| TEND\|DrugBank \| 17139284\|17016423 \| \| 1301 \| PDE4B \| Ambiguous \| PDE4B \| ROFLUMILAST \| inhibitor \| TdgClinicalTrial\|GuideToPharmacologyInteractions\|ChemblInteractions\| DrugBank \| 17726343 \| \| 1301 \| PDE4B \| Ambiguous \| PDE4B \| CILOMILAST \| inhibitor \| DrugBank \| 17155857 \| \| 1301 \| PDE4B \| Ambiguous \| PDE4B \| (-)-ROLIPRAM \| inhibitor \| DrugBank \| 11752352\|17139284\|17016423 \| \| 1301 \| PDE4B \| Ambiguous \| PDE4B \| IBUDILAST \| inhibitor \| TdgClinicalTrial\|GuideToPharmacologyInteractions\| DrugBank \| 16313925 \| \| 1301 \| PDE4B \| Ambiguous \| PDE4B \| APREMILAST \| antagonist\|inhibitor \| TdgClinicalTrial\|GuideToPharmacologyInteractions\|ChemblInteractions\|DrugBank \| 25864487\|17352685 \| \| 1301 \| PDE4B \| Ambiguous \| PDE4B \| PROPOXYPHENE \| inhibitor \| GuideToPharmacologyInteractions \|  \| \| 1301 \| PDE4B \| Ambiguous \| PDE4B \| CDP840 \| inhibitor \| GuideToPharmacologyInteractions \|  \| \| 1301 \| PDE4B \| Ambiguous \| PDE4B \| CRISABOROLE \| inhibitor \| TdgClinicalTrial\|GuideToPharmacologyInteractions\|ChemblInteractions\|DrugBank \|  \| \| 1301 \| PDE4B \| Ambiguous \| PDE4B \| SODIUM PHENYLBUTYRATE \| inhibitor \| GuideToPharmacologyInteractions \|  \| \| 1301 \| PDE4B \| Ambiguous \| PDE4B \| ROLIPRAM \| inhibitor \| GuideToPharmacologyInteractions\| DrugBank \| 10592235\|17139284\|17016423 \| \| 1301 \| PDE4B \| Ambiguous \| PDE4B \| CHEMBL1232082 \| inhibitor \| GuideToPharmacology Interactions \|  \| \| 1301 \| PDE4B \| Ambiguous \| PDE4B \| DIPYRIDAMOLE \| inhibitor \| ChemblInteractions \|  \| \| 1301 \| PDE4B \| Ambiguous \| PDE4B \| THEOPHYLLINE SODIUM GLYCINATE \| inhibitor \| ChemblInteractions \|  \| \| 1301 \| PDE4B \| Ambiguous \| PDE4B \| FLAVOXATE HYDROCHLORIDE \| inhibitor \| ChemblInteractions \|  \| \| 1301 \| PDE4B \| Ambiguous \| PDE4B \| AMINOPHYLLINE \| inhibitor \| ChemblInteractions \|  \| \| 1301 \| PDE4B \| Ambiguous \| PDE4B \| OXTRIPHYLLINE \| inhibitor \| ChemblInteractions \|  \| \| 1301 \| PDE4B \| Ambiguous \| PDE4B \| AMLEXANOX \| inhibitor \| ChemblInteractions \|  \| \| 1301 \| PDE4B \| Ambiguous \| PDE4B \| CHEMBL74078 \|  \| DrugBank \| 10592235 \| \| 1301 \| PDE4B \| Ambiguous \| PDE4B \| CHEMBL521203 \|  \| DrugBank \| 10592235 \| \| 1301 \| PDE4B \| Ambiguous \| PDE4B \| CAFFEINE \|  \| TdgClinicalTrial\| TEND \|  \| \| 1301 \| PDE4B \| Ambiguous \| PDE4B \| CHEMBL1230617 \|  \| DrugBank \| 17139284\|17016423 \| \| 1301 \| PDE4B \| Ambiguous \| PDE4B \| RESVERATROL \|  \| TdgClinicalTrial \|  \| \| 1301 \| PDE4B \| Ambiguous \| PDE4B \| [R]-Mesopram \|  \| DrugBank \| 10592235\|17139284\|17016423 \| \| 1301 \| PDE4B \| Ambiguous \| PDE4B \| CHEMBL519827 \|  \| DrugBank \| 10592235\|17139284\|17016423 \| \| 1301 \| PDE4B \| Ambiguous \| PDE4B \| ETAZOLATE \|  \| TdgClinicalTrial \|  \| \| 1301 \| PDE4B \| Ambiguous \| PDE4B \| FILAMINAST \|  \| DrugBank \| 10592235\|17139284\|17016423 \| \| 1301 \| PDE4B \| Ambiguous \| PDE4B \| PICLAMILAST \|  \| DrugBank \| 10592235\|15765929 \| \| 7058 \| THBS2 \| Definite \| THBS2 \| BEVACIZUMAB \|  \| NCI \| 16365183 \| \| 7058 \| THBS2 \| Definite \| THBS2 \| CORTICOTROPIN \|  \| NCI \| 8698834 \| \| 54107 \| POLE3 \| Definite \| POLE3 \| CLADRIBINE \| inhibitor \| DrugBank \| 19576186 \| \| 6342 \| KIT \| Ambiguous \| KIT \| SU-14813 \| inhibitor \| GuideToPharmacologyInteractions\|ChemblInteractions \|  \| \| 6342 \| KIT \| Ambiguous \| KIT \| SU-014813 \| inhibitor \| ChemblInteractions \|  \| \| 6342 \| KIT \| Ambiguous \| KIT \| FORETINIB \| inhibitor \| ChemblInteractions \|  \| \| 6342 \| KIT \| Ambiguous \| KIT \| TANDUTINIB \| inhibitor \| MyCancerGenome\|GuideToPharmacologyInteractions\|ChemblInteractions \|  \| \| 6342 \| KIT \| Ambiguous \| KIT \| PLX-3397 \| inhibitor \| MyCancerGenome\|CKB\|GuideToPharmacologyInteractions\|ChemblInteractions\|CancerCommons \| 24583793 \| \| 6342 \| KIT \| Ambiguous \| KIT \| XL-999 \| inhibitor \| TdgClinicalTrial\|ChemblInteractions \|  \| \| 6342 \| KIT \| Ambiguous \| KIT \| SEMAXANIB \| inhibitor \| GuideToPharmacologyInteractions\|ChemblInteractions \|  \| \| 6342 \| KIT \| Ambiguous \| KIT \| MASITINIB \| inhibitor \| MyCancerGenome\|TdgClinicalTrial\|GuideToPharmacologyInteractions\|ChemblInteractions\|CancerCommons\|MyCancerGenomeClinicalTrial \|  \| \| 6342 \| KIT \| Ambiguous \| KIT \| XL-820 \| inhibitor \| TALC\|MyCancerGenome\|TdgClinicalTrial\|ChemblInteractions\|DrugBank \|  \| \| 6342 \| KIT \| Ambiguous \| KIT \| SORAFENIB \| antagonist\|inhibitor \| TALC\|MyCancerGenome\|TdgClinicalTrial\|OncoKB\|CKB\|GuideToPharmacologyInteractions\|CGI\|CIViC\|TEND\|DrugBank\|DoCM \| 19139124\|18936790\|24317392\|19467857\|17229632\|17102120\|22665524\|21456006\|22357254\|17419150\|17545544\|22357255\|22270258\|17178882\|19671763\|20372153\|17699867\|19461405\|20571495\|25592632\|23140824\|25363205\|17272980\|20970876 \| \| 6342 \| KIT \| Ambiguous \| KIT \| TELATINIB \| inhibitor \| TALC\|MyCancerGenome\|Chembl  Interactions \|  \| \| 6342 \| KIT \| Ambiguous \| KIT \| DASATINIB \| antagonist\|inhibitor \| TALC\|TdgClinicalTrial\|OncoKB\|CKB\|ChemblInteractions\|CGI\|CIViC\|TEND\|DrugBank\|DoCM\|CancerCommons\|MyCancerGenomeClinicalTrial \| 21689725\|24045550\|19467857\|19164557\|15972446\|16384925\|18024392\|15685537\|17351742\|23149070\|25594040\|18986703\|21953054\|21456006\|17372901\|17419150\|22504184\|22357255\|22270258\|16912224\|18559612\|23714533\|19671763\|25157968\|17699867\|22932406\|17259998\|21642685\|16434489\|16397263\|23582185\|23140824\|16731599 \| \| 6342 \| KIT \| Ambiguous \| KIT \| SUNITINIB \| inhibitor \| TALC\|MyCancerGenome\|TdgClinicalTrial\|OncoKB\|CKB\|GuideToPharmacologyInteractions\|CGI\|CIViC\|TEND\|DrugBank\|DoCM\|MyCancerGenomeClinicalTrial\|TTD \| 19282169\|25641662\|19164557\|17545799\|16098458\|12181401\|18235121\|19861435\|23177515\|25594040\|21969494\|17367763\|16638875\|21690468\|23375402\|22357254\|17046465\|18955458\|22261812\|25239608\|14753710\|22439647\|11752352\|18421059\|19461405\|21642685\|12748309\|15451219\|20571495\|25592632\|23582185\|26772734\|12873999 \| \| 6342 \| KIT \| Ambiguous \| KIT \| NILOTINIB \| antagonist\|inhibitor \| OncoKB\|CKB\|CGI\|CIViC\|DrugBank\|CancerCommons\|MyCancerGenomeClinicalTrial\|TTD \| 28720666\|28843487\|19467857\|20442311\|25594040\|21456006\|17372901\|17419150\|25209843\|22119758\|22357255\|28327988\|22068222\|25695690\|22270258\|19671763\|17699867\|23582185\|23140824 \| \| 6342 \| KIT \| Ambiguous \| KIT \| PAZOPANIB \| inhibitor \| TALC\|MyCancerGenome\|GuideToPharmacologyInteractions\|DrugBank\|MyCancerGenomeClinicalTrial\|TTD \| 17288876 \| \| 6342 \| KIT \| Ambiguous \| KIT \| REGORAFENIB \| inhibitor \| TALC\|MyCancerGenome\|OncoKB\|CKB\|ChemblInteractions\|CGI\|DrugBank\|MyCancerGenomeClinicalTrial \| 21170960\|19282169\|25641662\|27371698\|16098458\|12181401\|18235121\|23177515\|17046465\|25239608\|15451219 \| \| 6342 \| KIT \| Ambiguous \| KIT \| PONATINIB \| inhibitor \| CKB\|CGI\|DrugBank \| 24552773\|10517\|21482694\|22301675\|25239608\|10535\|23539538\|2015 \| \| 6342 \| KIT \| Ambiguous \| KIT \| AKN-028 \| inhibitor \| GuideToPharmacologyInteractions \|  \| \| 6342 \| KIT \| Ambiguous \| KIT \| AST-487 \| inhibitor \| GuideToPharmacologyInteractions \|  \| \| 6342 \| KIT \| Ambiguous \| KIT \| CEDIRANIB \| inhibitor \| TALC\|GuideToPharmacologyInteractions\|ChemblInteractions \|  \| \| 6342 \| KIT \| Ambiguous \| KIT \| PHENOBARBITAL \| inhibitor \| GuideToPharmacologyInteractions \|  \| \| 6342 \| KIT \| Ambiguous \| KIT \| CRENOLANIB \| inhibitor \| GuideToPharmacologyInteractions \|  \| \| 6342 \| KIT \| Ambiguous \| KIT \| DIPHENIDOL \| inhibitor \| CKB\|GuideToPharmacologyInteractions \|  \| \| 6342 \| KIT \| Ambiguous \| KIT \| DOVITINIB \| inhibitor \| ClearityFoundationClinicalTrial\|GuideToPharmacologyInteractions\|ChemblInteractions \|  \| \| 6342 \| KIT \| Ambiguous \| KIT \| Famitinib \| inhibitor \| GuideToPharmacologyInteractions\|ChemblInteractions \|  \| \| 6342 \| KIT \| Ambiguous \| KIT \| CHEMBL406375 \| inhibitor \| GuideToPharmacologyInteractions \|  \| \| 6342 \| KIT \| Ambiguous \| KIT \| JNJ-40346527 \| inhibitor \| GuideToPharmacologyInteractions \|  \| \| 6342 \| KIT \| Ambiguous \| KIT \| CHEMBL1908396 \| inhibitor \| GuideToPharmacologyInteractions \|  \| \| 6342 \| KIT \| Ambiguous \| KIT \| LINIFANIB \| inhibitor \| TdgClinicalTrial\|GuideToPharmacologyInteractions\|DrugBank \|  \| \| 6342 \| KIT \| Ambiguous \| KIT \| LUCITANIB \| inhibitor \| GuideToPharmacologyInteractions \|  \| \| 6342 \| KIT \| Ambiguous \| KIT \| OSI-930 \| inhibitor \| GuideToPharmacologyInteractions\|ChemblInteractions\|DrugBank\|TTD \|  \| \| 6342 \| KIT \| Ambiguous \| KIT \| QUIZARTINIB \| inhibitor \| CKB\|GuideToPharmacologyInteractions\|ChemblInteractions\|DoCM \| 23497317\|21689725\|24045550\|19164557\|15972446\|16384925\|18024392\|18986703\|22504184\|16912224\|23714533\|25157968\|17259998\|23582185\|16731599 \| \| 6342 \| KIT \| Ambiguous \| KIT \| SU-11652 \| inhibitor \| GuideToPharmacologyInteractions \|  \| \| 6342 \| KIT \| Ambiguous \| KIT \| PAZOPANIB HYDROCHLORIDE \| inhibitor \| ChemblInteractions\|TTD \|  \| \| 6342 \| KIT \| Ambiguous \| KIT \| CM-082 \| inhibitor \| ChemblInteractions \|  \| \| 6342 \| KIT \| Ambiguous \| KIT \| ENMD-2076 \| inhibitor \| TdgClinicalTrial\|ChemblInteractions \|  \| \| 6342 \| KIT \| Ambiguous \| KIT \| AMUVATINIB \| inhibitor \| TALC\|MyCancerGenome\|TdgClinicalTrial\|ChemblInteractions\|DrugBank\|TTD \| 17325667 \| \| 6342 \| KIT \| Ambiguous \| KIT \| MIDOSTAURIN \| inhibitor \| MyCancerGenome\|CKB\|ChemblInteractions\|CIViC\|MyCancerGenomeClinicalTrial\|FDA \| 25209843\|27355533 \| \| 6342 \| KIT \| Ambiguous \| KIT \| VATALANIB \| inhibitor \| TALC\|TdgClinicalTrial\|ChemblInteractions \|  \| \| 6342 \| KIT \| Ambiguous \| KIT \| MOTESANIB \| inhibitor \| TALC\|MyCancerGenome\|TdgClinicalTrial\|CKB\|ChemblInteractions\|TTD \| 20633291 \| \| 6342 \| KIT \| Ambiguous \| KIT \| SORAFENIB TOSYLATE \| inhibitor \| ChemblInteractions \|  \| \| 6342 \| KIT \| Ambiguous \| KIT \| SUNITINIB MALATE \| inhibitor \| ChemblInteractions\|TTD \|  \| \| 6342 \| KIT \| Ambiguous \| KIT \| IMATINIB MESYLATE \| inhibitor \| ChemblInteractions\|MyCancerGenomeClinicalTrial \|  \| \| 6342 \| KIT \| Ambiguous \| KIT \| AXITINIB \| inhibitor \| TALC\|MyCancerGenome\|TdgClinicalTrial\|CancerCommons \|  \| \| 6342 \| KIT \| Ambiguous \| KIT \| CABOZANTINIB \| inhibitor \| MyCancerGenome\|CKB \| 24205792\|25836719\|27777285 \| \| 6342 \| KIT \| Ambiguous \| KIT \| IMATINIB \| inhibitor \| TALC\|MyCancerGenome\|TdgClinicalTrial\|OncoKB\|CKB\|CGI\|CIViC\|TEND\|DoCM\|TTD\|FDA \| 18795925\|15790786\|9438854\|21159146\|21689725\|19282169\|20088873\|26316776\|24045550\|27370604\|25641662\|18936790\|24317392\|25673643\|26687836\|7530509\|15236194\|19164557\|22114577\|15201427\|15972446\|16384925\|16098458\|12181401\|18024392\|27980106\|12481435\|15685537\|25015329\|18235121\|28611108\|19861435\|23177515\|11276010\|25594040\|21969494\|16638875\|28196207\|18986703\|21953054\|16143141\|21690468\|24419427\|23375402\|18980976\|22357254\|17372901\|17046465\|22504184\|25003536\|18955458\|23567324\|24687822\|22261812\|16908931\|16624552\|19175693\|16912224\|25239608\|15930355\|18421059\|19812602\|18955451\|23775962\|23714533\|19671763\|25157968\|20372153\|16046538\|17699867\|22932406\|14645423\|17363509\|17259998\|12374669\|21642685\|18751412\|16954519\|15451219\|16015387\|16462496\|16751810\|23582185\|27777285\|22355224\|28334365\|16731599 \| \| 6342 \| KIT \| Ambiguous \| KIT \| MOTESANIB DIPHOSPHATE \| inhibitor \| TTD \|  \| \| 6342 \| KIT \| Ambiguous \| KIT \| Anlotinib \| inhibitor \| ChemblInteractions \|  \| \| 6342 \| KIT \| Ambiguous \| KIT \| PEMBROLIZUMAB \|  \| CKB \| 28514312 \| \| 6342 \| KIT \| Ambiguous \| KIT \| CHEMBL2325503 \|  \| CKB \| 28611108 \| \| 6342 \| KIT \| Ambiguous \| KIT \| CYTARABINE \|  \| CKB \| 18986703 \| \| 6342 \| KIT \| Ambiguous \| KIT \| INFIGRATINIB \|  \| CKB \| 25673643 \| \| 6342 \| KIT \| Ambiguous \| KIT \| TRAMETINIB \|  \| CKB \| 28514312 \| \| 6342 \| KIT \| Ambiguous \| KIT \| CHEMBL286939 \|  \| DrugBank \| 17139284\|17016423 \| \| 6342 \| KIT \| Ambiguous \| KIT \| PD-0325901 \|  \| CKB \| 26936919 \| \| 6342 \| KIT \| Ambiguous \| KIT \| MK-2206 \|  \| CKB \| 27370604 \| \| 6342 \| KIT \| Ambiguous \| KIT \| LENVATINIB \|  \| TdgClinicalTrial \|  \| \| 6342 \| KIT \| Ambiguous \| KIT \| BEVACIZUMAB \|  \| CKB \| 25363205 \| \| 9265 \| CYTH3 \| Definite \| CYTH3 \| CHEMBL23552 \|  \| DrugBank \| 10592235\|17139284\|17016423 \| \| 9265 \| CYTH3 \| Definite \| CYTH3 \| CHEMBL1207374 \|  \| DrugBank \| 10592235\|17139284\|17016423 \| \| 65263 \| PYCR3 \| Definite \| PYCR3 \| L-PROLINE \|  \| DrugBank \| 10592235\|17139284\|17016423 \| \| 10630 \| CCL4 \| Ambiguous \| CCL4 \| CYCLOSPORINE \|  \| NCI \| 15153516 \| \| 10630 \| CCL4 \| Ambiguous \| CCL4 \| EPOETIN ALFA \|  \| NCI \| 17119331 \| \| 34 \| ACADM \| Ambiguous \| ACADM \| FLAVIN ADENINE DINUCLEOTIDE \|  \| DrugBank \| 10592235\|17139284\|17016423 \| \| 34 \| ACADM \| Ambiguous \| ACADM \| Octanoyl-CoA \|  \| DrugBank \| 10592235\|17139284\|17016423 \| \| 34 \| AGTR1 \| Ambiguous \| AGTR1 \| VALSARTAN \| antagonist \| TdgClinicalTrial\|GuideToPharmacologyInteractions\|ChemblInteractions\|TEND\|DrugBank\|TTD \| 8242249\|12460705\|8577935\|15579516\|12023686\|11752352 \| \| 34 \| AGTR1 \| Ambiguous \| AGTR1 \| TELMISARTAN \| antagonist \| TdgClinicalTrial\|GuideToPharmacologyInteractions\|ChemblInteractions\|TEND\|DrugBank\|TTD \| 9878991\|11408526\|15617852\|19147680\|11558835\|16938288\|15498586\|10067800\|11444497\|20448797\|9259062\|11752352\|17691961\|18580862 \| \| 34 \| AGTR1 \| Ambiguous \| AGTR1 \| IRBESARTAN \| antagonist \| PharmGKB\|TdgClinicalTrial\|GuideToPharmacologyInteractions\|ChemblInteractions\|TEND\|DrugBank\|TTD \| 7843749\|10373224\|10822210\|15101793\|14716205\|18627212\|11752352\|10069682\|10075381\|11486244\|15030294\|17408613\|10082498 \| \| 34 \| AGTR1 \| Ambiguous \| AGTR1 \| FORASARTAN \| antagonist \| TdgClinicalTrial\|TEND\|DrugBank\|TTD \| 8981065\|9156352\|11569611\|11752352 \| \| 34 \| AGTR1 \| Ambiguous \| AGTR1 \| SAPRISARTAN POTASSIUM \| antagonist \| TdgClinicalTrial\|TEND\|DrugBank\|TTD \| 10579749\|11752352 \| \| 34 \| AGTR1 \| Ambiguous \| AGTR1 \| TASOSARTAN \| antagonist \| TdgClinicalTrial\|GuideToPharmacologyInteractions\|TEND\|DrugBank\|TTD \| 11683476\|12113820\|11752352\|11046101 \| \| 34 \| AGTR1 \| Ambiguous \| AGTR1 \| EPROSARTAN MESYLATE \| antagonist \| ChemblInteractions\|TTD \|  \| \| 34 \| AGTR1 \| Ambiguous \| AGTR1 \| OLMESARTAN \| antagonist \| TdgClinicalTrial\|GuideToPharmacologyInteractions\|ChemblInteractions\|TEND\|TTD \|  \| \| 34 \| AGTR1 \| Ambiguous \| AGTR1 \| OLMESARTAN MEDOXOMIL \| antagonist \| ChemblInteractions\|TTD \|  \| \| 34 \| AGTR1 \| Ambiguous \| AGTR1 \| CANDESARTAN \| antagonist \| TdgClinicalTrial\|GuideToPharmacologyInteractions\|TEND\|TTD \|  \| \| 34 \| AGTR1 \| Ambiguous \| AGTR1 \| EPROSARTAN \| antagonist \| TdgClinicalTrial\|GuideToPharmacologyInteractions\|TEND\|TTD \|  \| \| 34 \| AGTR1 \| Ambiguous \| AGTR1 \| ANGIOTENSIN II \| agonist \| GuideToPharmacologyInteractions \|  \| \| 34 \| AGTR1 \| Ambiguous \| AGTR1 \| ANGIOTENSIN III \| agonist \| GuideToPharmacologyInteractions \|  \| \| 34 \| AGTR1 \| Ambiguous \| AGTR1 \| CHEMBL288174 \| agonist \| GuideToPharmacologyInteractions \|  \| \| 34 \| AGTR1 \| Ambiguous \| AGTR1 \| ANGIOTENSIN IV \| agonist \| GuideToPharmacologyInteractions \|  \| \| 34 \| AGTR1 \| Ambiguous \| AGTR1 \| AZILSARTAN \| antagonist \| TdgClinicalTrial\|GuideToPharmacologyInteractions\|ChemblInteractions \|  \| \| 34 \| AGTR1 \| Ambiguous \| AGTR1 \| LOSARTAN \| antagonist \| TdgClinicalTrial\|GuideToPharmacologyInteractions\|TEND \|  \| \| 34 \| AGTR1 \| Ambiguous \| AGTR1 \| CHEMBL345132 \| antagonist \| GuideToPharmacologyInteractions \|  \| \| 34 \| AGTR1 \| Ambiguous \| AGTR1 \| CHEMBL344662 \| antagonist \| GuideToPharmacologyInteractions \|  \| \| 34 \| AGTR1 \| Ambiguous \| AGTR1 \| METYRAPONE \| antagonist \| GuideToPharmacologyInteractions \|  \| \| 34 \| AGTR1 \| Ambiguous \| AGTR1 \| MEXILETINE \| antagonist \| GuideToPharmacologyInteractions \|  \| \| 34 \| AGTR1 \| Ambiguous \| AGTR1 \| SPARSENTAN \| antagonist \| TdgClinicalTrial\|GuideToPharmacologyInteractions\|ChemblInteractions \|  \| \| 34 \| AGTR1 \| Ambiguous \| AGTR1 \| PRATOSARTAN \| antagonist \| ChemblInteractions \|  \| \| 34 \| AGTR1 \| Ambiguous \| AGTR1 \| AZILSARTAN KAMEDOXOMIL \| antagonist \| ChemblInteractions \|  \| \| 34 \| AGTR1 \| Ambiguous \| AGTR1 \| LOSARTAN POTASSIUM \| antagonist \| ChemblInteractions \|  \| \| 34 \| AGTR1 \| Ambiguous \| AGTR1 \| SARALASIN ACETATE \| antagonist \| ChemblInteractions \|  \| \| 34 \| AGTR1 \| Ambiguous \| AGTR1 \| CANDESARTAN CILEXETIL \| antagonist \| ChemblInteractions \|  \| \| 34 \| AGTR1 \| Ambiguous \| AGTR1 \| VOLANESORSEN SODIUM \|  \| NCI \| 10100098 \| \| 34 \| AGTR1 \| Ambiguous \| AGTR1 \| ATORVASTATIN \|  \| NCI \| 11179461 \| \| 34 \| AGTR1 \| Ambiguous \| AGTR1 \| CYCLOSPORINE \|  \| NCI \| 17477024 \| \| 34 \| AGTR1 \| Ambiguous \| AGTR1 \| INDOMETHACIN \|  \| NCI \| 9357777 \| \| 34 \| AGTR1 \| Ambiguous \| AGTR1 \| IDOXIFENE \|  \| NCI \| 11588112 \| \| 34 \| AGTR1 \| Ambiguous \| AGTR1 \| HYDROGEN PEROXIDE \|  \| NCI \| 18006461 \| \| 34 \| AGTR1 \| Ambiguous \| AGTR1 \| GENISTEIN \|  \| NCI \| 7476882 \| \| 34 \| AGTR1 \| Ambiguous \| AGTR1 \| HYDROCHLOROTHIAZIDE \|  \| PharmGKB \|  \| \| 34 \| AGTR1 \| Ambiguous \| AGTR1 \| STANOLONE \|  \| NCI \| 16990489\|16482568 \| \| 34 \| AGTR1 \| Ambiguous \| AGTR1 \| LEVODOPA \|  \| NCI \| 9458818 \| \| 34 \| AGTR1 \| Ambiguous \| AGTR1 \| DEXAMETHASONE \|  \| NCI \| 16482568 \| \| 34 \| AGTR1 \| Ambiguous \| AGTR1 \| INTERFERON GAMA-1B \|  \| NCI \| 9231818 \| \| 34 \| AGTR1 \| Ambiguous \| AGTR1 \| ASCORBATE \|  \| NCI \| 18091746 \| \| 34 \| MGMT \| Ambiguous \| MGMT \| CISPLATIN (CHEMBL2068237) \|  \| NCI \| 16043385 \| \| 34 \| MGMT \| Ambiguous \| MGMT \| CYSTEINE \|  \| DrugBank \| 16950796 \| \| 34 \| MGMT \| Ambiguous \| MGMT \| THIOGUANINE \|  \| NCI \| 1638685 \| \| 34 \| MGMT \| Ambiguous \| MGMT \| EXATECAN MESYLATE \|  \| NCI \| 11802813 \| \| 34 \| MGMT \| Ambiguous \| MGMT \| NIMUSTINE HYDROCHLORIDE \|  \| NCI \| 1518162 \| \| 34 \| MGMT \| Ambiguous \| MGMT \| CAMPTOTHECIN \|  \| NCI \| 16258022 \| \| 34 \| MGMT \| Ambiguous \| MGMT \| DACARBAZINE \|  \| NCI \| 12168066 \| \| 34 \| MGMT \| Ambiguous \| MGMT \| ETHYL NITROSOUREA \|  \| NCI \| 1727384 \| \| 34 \| MGMT \| Ambiguous \| MGMT \| TEMOZOLOMIDE \|  \| NCI\|CIViC \| 15486188\|25910840\|15758010\|21365007\|17442989\|21331613 \| \| 34 \| MGMT \| Ambiguous \| MGMT \| HYCANTHONE \|  \| NCI \| 9609323 \| \| 34 \| MGMT \| Ambiguous \| MGMT \| IFOSFAMIDE \|  \| NCI \| 8621235 \| \| 34 \| MGMT \| Ambiguous \| MGMT \| CHLORAMBUCIL \|  \| NCI \| 8621235 \| \| 34 \| MGMT \| Ambiguous \| MGMT \| GENISTEIN \|  \| NCI \| 16203797 \| \| 34 \| MGMT \| Ambiguous \| MGMT \| N-METHYL-N-NITROSUREA \|  \| NCI \| 9192819\|10469609\|10439048 \| \| 34 \| MGMT \| Ambiguous \| MGMT \| ETHACRYNIC ACID \|  \| NCI \| 9155531 \| \| 34 \| MGMT \| Ambiguous \| MGMT \| CHEMBL394875 \|  \| DrugBank \| 17139284\|17016423 \| \| 34 \| MGMT \| Ambiguous \| MGMT \| AZACITIDINE \|  \| NCI \| 7511991 \| \| 34 \| MGMT \| Ambiguous \| MGMT \| MELPHALAN \|  \| NCI \| 8621235 \| \| 34 \| MGMT \| Ambiguous \| MGMT \| LOMUSTINE \|  \| NCI \| 9393761 \| \| 34 \| MGMT \| Ambiguous \| MGMT \| CARMUSTINE \|  \| CIViC \| 11070098 \| \| 34 \| MGMT \| Ambiguous \| MGMT \| MAFOSFAMIDE \|  \| NCI \| 8621235 \| \| 34 \| MGMT \| Ambiguous \| MGMT \| BENZYLCYSTEINE \|  \| DrugBank \| 17139284\|17016423 \| \| 34 \| MGMT \| Ambiguous \| MGMT \| IRINOTECAN \|  \| NCI \| 11802813 \| \| 34 \| MGMT \| Ambiguous \| MGMT \| FOTEMUSTINE \|  \| NCI \| 11279615 \| \| 1948 \| BTK \| Ambiguous \| BTK \| ACALABRUTINIB \| inhibitor \| GuideToPharmacologyInteractions \|  \| \| 1948 \| BTK \| Ambiguous \| BTK \| ISONIAZID \| inhibitor \| GuideToPharmacologyInteractions \|  \| \| 1948 \| BTK \| Ambiguous \| BTK \| IBRUTINIB \| inhibitor \| MyCancerGenome\|TdgClinicalTrial\|GuideToPharmacologyInteractions\|ChemblInteractions\|CGI\|CIViC\|DrugBank\|  DoCM \| 26182309\|27626698\|24869598\|25802231\|27199251 \| \| 1948 \| BTK \| Ambiguous \| BTK \| CHEMBL228043 \| inhibitor \| GuideToPharmacologyInteractions \|  \| \| 1948 \| BTK \| Ambiguous \| BTK \| ONO-4059 \| inhibitor \| GuideToPharmacologyInteractions\|ChemblInteractions \|  \| \| 1948 \| BTK \| Ambiguous \| BTK \| SPEBRUTINIB \| inhibitor \| GuideToPharmacologyInteractions \|  \| \| 1948 \| BTK \| Ambiguous \| BTK \| CHEMBL1229592 \| inhibitor \| GuideToPharmacologyInteractions \|  \| \| 1948 \| BTK \| Ambiguous \| BTK \| SPEBRUTINIB BESYLATE \| inhibitor \| ChemblInteractions \|  \| \| 1948 \| BTK \| Ambiguous \| BTK \| HM-71224 \| inhibitor \| ChemblInteractions \|  \| \| 1948 \| BTK \| Ambiguous \| BTK \| MSC-2364447 \| inhibitor \| ChemblInteractions \|  \| \| 1948 \| BTK \| Ambiguous \| BTK \| XL-418 \|  \| DrugBank \|  \| \| 1948 \| BTK \| Ambiguous \| BTK \| CHEMBL23552 \|  \| DrugBank \| 17139284\|17016423 \| |


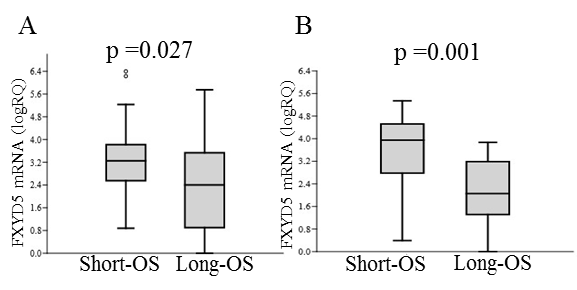


**Figure S1.** Box plots showing FXYD5 mRNA levels in HGSOC patients by RT-qPCR. Significant FXYD5 overexpression in short-term survivors (short-OS) compared to long-term ones (long-OS) was confirmed (FC=1.96) in the training set, (A), and further validated in a second independent cohort (FC=2.68) (B).


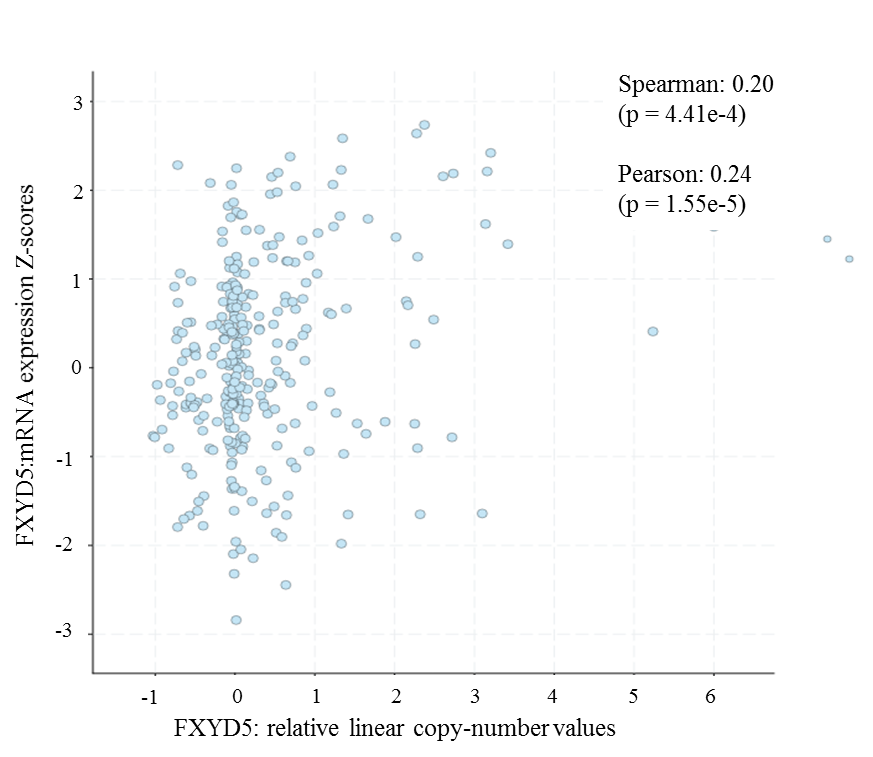


**Figure S2.** Correlation between FXYD5 expression levels and copy number values in HGSOC patients determined by the cBioPortal tool (https://www.cbioportal.org/). Relative copy number values: -1 = shallow loss, possible heterozygous deletion; 0 = diploid; 1 = low-level gain (a few additional copies, often broad); 2 = high-level amplification (more copies, often focal).


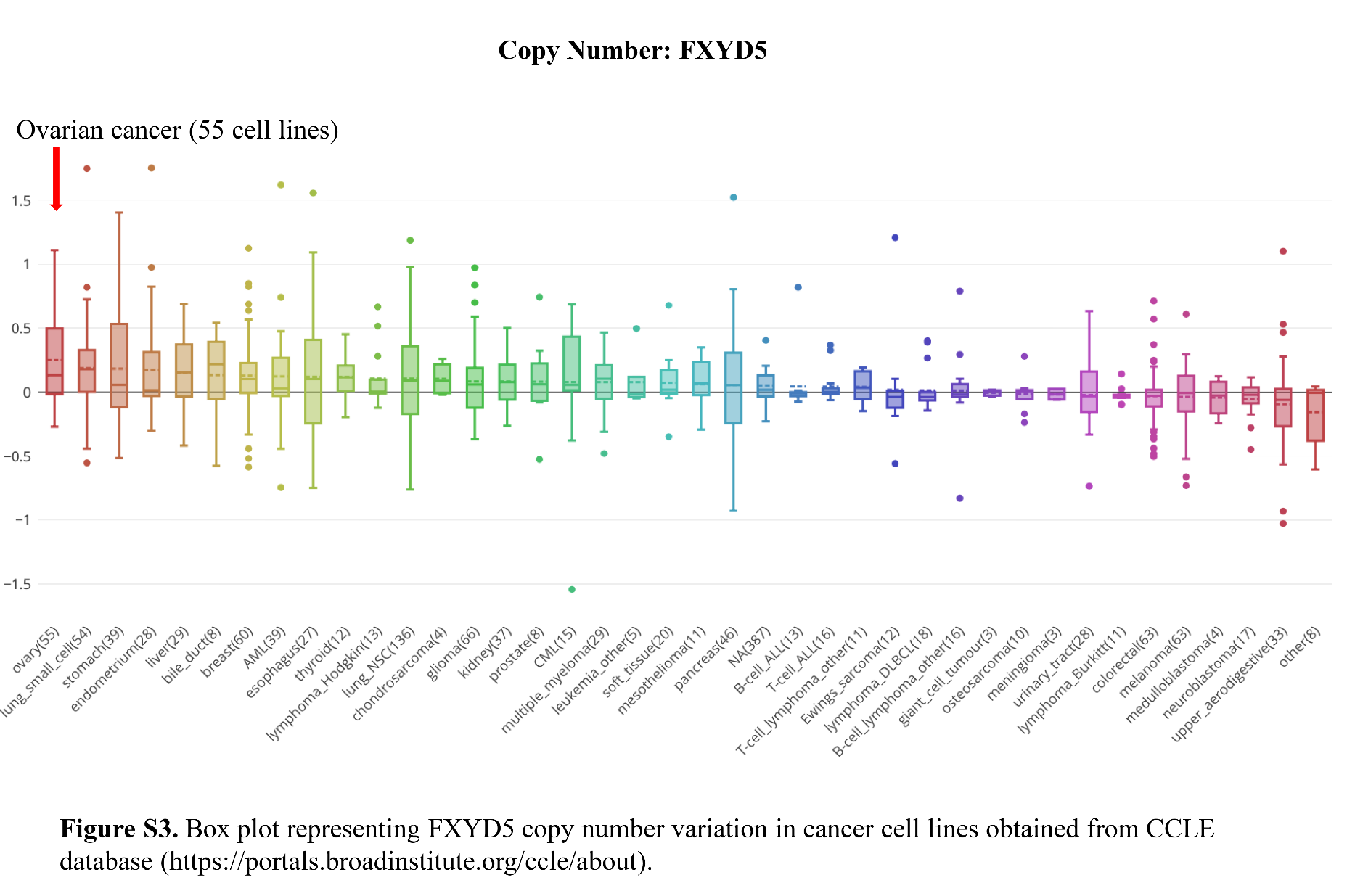

Supplement: Supplementary file 1 — Supplementary materials [file 41416_2019_553_MOESM1_ESM.docx]
